# Photosynthetic Carbon Fixation and Sucrose Metabolism Supplemented by Weighted Gene Co-expression Network Analysis in Response to Water Stress in Rice With Overlapping Growth Stages

**DOI:** 10.3389/fpls.2022.864605

**Published:** 2022-04-21

**Authors:** Xinpeng Wang, Hualong Liu, Di Zhang, Detang Zou, Jingguo Wang, Hongliang Zheng, Yan Jia, Zhaojun Qu, Bin Sun, Hongwei Zhao

**Affiliations:** Key Laboratory of Germplasm Enhancement, Physiology and Ecology of Food Crops in Cold Region, Ministry of Education, Northeast Agricultural University, Harbin, China

**Keywords:** overlapping growth stages, drought stress, sucrose metabolism, carbon fixation, transcriptional analysis, weighted gene co-expression network analysis (WGCNA)

## Abstract

Drought stress at jointing and booting phases of plant development directly affects plant growth and productivity in rice. Limited by natural factors, the jointing and booting stages in rice varieties are known to overlap in high-latitude areas that are more sensitive to water deficit. However, the regulation of photosynthetic carbon fixation and sucrose metabolism in rice leaves under different degrees of drought stress remains unclear. In this study, rice plants were subjected to three degrees of drought stress (−10, −25, −and 40 kPa) for 15 days during the jointing-booting stage, we investigated photosynthetic carbon sequestration and sucrose metabolism pathways in rice leaves and analyzed key genes and regulatory networks using transcriptome sequencing in 2016. And we investigated the effects of drought stress on the growth periods of rice with overlapping growth periods in 2016 and 2017. The results showed that short-term drought stress promoted photosynthetic carbon fixation. However, ribulose-1,5-bisphosphate carboxylase/oxygenase (RuBisCO) activity significantly decreased, resulting in a significant decrease in photosynthetic rate. Drought stress increased the maximum activity of fructose-1,6-bisphosphate aldolase (FBA). FBA maintains the necessary photosynthetic rate during drought stress and provides a material base after the resumption of irrigation in the form of controlling the content of its reaction product triose phosphate. Drought stress significantly affected the activities of sucrose synthase (SuSase) and sucrose phosphate synthase (SPS). Vacuoles invertase (VIN) activity increased significantly, and the more severe the drought, the higher the VIN activity. Severe drought stress at the jointing-booting stage severely restricted the growth process of rice with overlapping growth stages and significantly delayed heading and anthesis stages. Transcriptome analysis showed that the number of differentially expressed genes was highest at 6–9 days after drought stress. Two invertase and four β-amylase genes with time-specific expression were involved in sucrose-starch metabolism in rice under drought stress. Combined with weighted gene co-expression network analysis, VIN and β-amylase genes up-regulated throughout drought stress were regulated by *OsbZIP04* and *OsWRKY62* transcription factors under drought stress. This study showed that any water deficit at the jointing-booting stage would have a serious effect on sucrose metabolism in leaves of rice with overlapping growth stages.

## Introduction

Drought has become the main cause of crop yield reductions worldwide. For example, China suffered an agricultural drought in 2016, and the affected area totalled 9,872.7 thousand hectares, according to the National Bureau of Statistics of China [NBSC] (2017). Given that a decrease in crop production caused by drought has the potential to cause significant economic disruption, the demand for the development of drought-tolerant crops is increasing (Todaka et al., 2015). Locally adapted drought stress tolerance traits are needed to achieve maximal crop yield potential (Greenham et al., 2017).

In 2020, the rice (*Oryza sativa* L.)-cultivated area totaled 30,080 thousand hectares in China, producing a yield of 211.9 million tons (NBSC). As one of the world’s most important cereals, rice is also the most water-intensive crop. Compared with other cereal crops, rice cultivars have been planted with zero soil water potential for thousands of years, which causes greater sensitivity to decreases in soil-water potential. Rice production requires large amounts of water. Three to five thousand liters of water are required for the production of 1 kg of rice seed, while other crops, such as maize or wheat, require less than half of that (Singh et al., 2002). Therefore, understanding the range of physiological changes initiated by drought stress is important for developing supportive measures to enhance drought resistance.

The drought response involves changing molecular, biochemical, and physiological mechanisms and morphology of plants (Chaves et al., 2009; Yousfi et al., 2016), and the duration and intensity of drought are of paramount importance in these responses. Severe or prolonged drought has been found to negatively affect leaf growth (Caldeira et al., 2014) by inhibiting cell proliferation and expansion (Baerenfaller et al., 2012). This is because drought inhibits photosynthesis and carbon metabolism (Hamilton et al., 2001), resulting in a lack of a sufficient carbon skeleton for cell proliferation. The mobilization of non-structural carbohydrates (NSC) is the basis of plant carbon metabolism. A large number of studies have found that drought increases the level of NSCs in the short term (Kozlowski and Pallardy, 2002), but a long period of drought forced NSC to be consumed for the maintenance of cellular survival, including respiratory metabolism and osmotic adjustment (Amthor and McCree, 1990). The role of sucrose has been the focus of attention among NSCs, not only providing nutrition for plant cells, but also as a signal molecule of many metabolic pathways (Couée et al., 2006; Bolouri-Moghaddam et al., 2010). Therefore, studying the influence of drought stress on sucrose metabolism is key to understanding the mechanism of carbon metabolism in response to drought.

Studies have shown that high levels of sucrose can enhance plant resistance (Lv et al., 2008). The role of sucrose in metabolism is far more than that in many cases, as sucrose signaling molecules involved in metabolic pathways form other substances, such as anthocyanins and fructans (Teng et al., 2005; Martínez-Noël et al., 2009). Sucrose is essential for the induction of fructosyltransferases (FT) genes and acts as a substrate for FT enzymes that synthesize fructose. It is obvious that the actual level of sucrose greatly determines the accumulation of fructans. The actual level of sucrose is determined by the balance between sucrose biosynthesis (sucrose phosphate synthase/sucrose phosphate phosphatase) and sucrose degradation (invertases, sucrose synthase). Thus, it can indirectly affect the synthesis of fructose and the growth and development of plants by affecting sucrose metabolism. Concerning the key enzymes involved in sucrose metabolism, contradictory data on drought effects have been reported. Some studies have found that SPS activity decreases or remains unchanged in maize, potato, soybean, and some other crops under drought stress (Vassey et al., 1991), while some data exhibited an increase in SPS activity in rice and wheat (Niedzwiedz-Siegien et al., 2004). Sucrose synthase (SuSase) and invertase (INV) are enzymes that catalyze the cleavage of sucrose in higher plants. SuSase is primarily involved in the biosynthesis of sucrose polymers, such as starch and cellulose, and the production of energy (ATP). Depending on the optimal pH, INV can be divided into three types (VIN, CWIN, and CIN), and its subcellular localization and function are different. VIN exists in the vacuoles of plant cells and catalyzes the hydrolysis of sucrose to hexose. It regulates the accumulation of sucrose in plant tissues and the utilization of sucrose in vacuoles.

Sucrose synthesis is based on the Calvin cycle. Fructose-bisphosphate aldolase (FBA) and ribulose-1,5-bisphosphate carboxylase/oxygenase (RuBisCO) are the key enzymes of the Calvin cycle, which is the main pathway for photosynthetic carbon fixation. The activity and expression of these two enzymes will change under drought stress. Most of the evidence on drought-induced changes indicates that the activity of RuBisCO and FBA declines during drought (Chaitanya et al., 2003). This indirectly affects the rate of sucrose synthesis.

Transcription levels under drought stress have been the focus of numerous plant species (Zhang et al., 2012; Smita et al., 2013). Many drought-responsive genes are time-sensitive (Wilkins et al., 2010; Dubois et al., 2017), so time effects must be considered in order to associate physiological changes with transcriptomics. The combined action of time and abiotic stress complicates the analysis of transcriptomics (Greenham and McClung, 2015). Scholars have found that the differences in transcriptomics caused by time factors are even greater than abiotic stresses (Wilkins et al., 2010; Greenham et al., 2017).

Network analysis is a powerful way to comprehensively analyze the meaningful differences among time, treatments, and development by providing a pathway structure (Priest et al., 2014; Gehan et al., 2015; Righetti et al., 2015). Weighted gene co-expression network analysis (WGCNA) has been widely applied in recent years to analyze the association of transcriptional changes with physiological differences in different periods. Here, we analyzed the transcriptome and physiological parameters of rice varieties with different drought tolerances during 12 days of drought stress using the WGCNA approach.

Rice in cold regions has a short growth period. The jointing and booting stages of these varieties overlap, and vegetative growth and reproductive growth occur simultaneously. There is also a high overlap between the heading and anthesis stages. The overlapping characteristics of growth stages also determine its extreme sensitivity to water during the transition period of the growth stage, especially at the jointing-booting stage. The kind of damage that water deficiency at the jointing-booting stage will cause photosynthetic capacity and the effects of different degrees of drought stress on photosynthetic carbon fixation and sucrose metabolism need to be investigated.

In this study, we selected two typical rice varieties in cold regions with overlapping growth stages. We set three different degrees of drought stress treatments at the jointing-booting stage: mild, moderate, and severe drought stress, to explore the differences with respect to the physiological and transcriptional characteristics in leaf photosynthesis and sucrose metabolism under different water deficit conditions.

## Materials and Methods

### Plant Material and Growth Conditions

**FIGURE 1:**
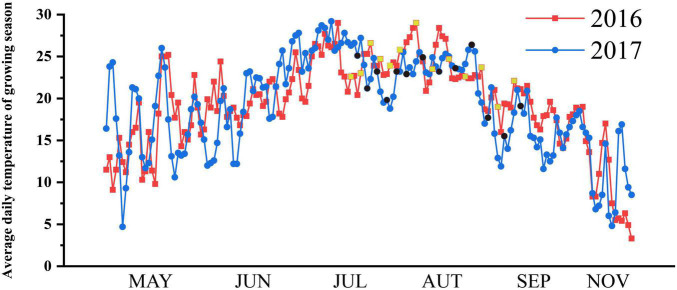
Average daily temperature (°C) of growing season in experimental site. The sampling dates were marked yellow squares (2016) and black circles (2017).

The research was conducted in the rain proof shelter of A’Cheng experimental site of the Northeast Agricultural University in Harbin City, China (126°40 E, 45°10 N) during the rice-growing season (April–September) in 2016 (field experiment) and 2017 (pot experiment). The frost-free period is short at high latitudes, and the suitable season for rice growth is only from May to October. The average daily temperature during the growing season at the experimental site is shown in Figure 1. Two typical *japonica* rice varieties *Oryza sativa* subsp. *Japonica* with overlapping growth periods, Songjing 6 (SJ6, 135 days growth period and 2500°C effective accumulated temperature) and Dongnong 425 (DN425, 140 days growth period and 2550°C effective accumulated temperature) were used as experimental materials. In mid-April of 2016 and 2017, rice seeds were sown in the seedbed and transplanted in mid-May with a hill spacing of 30 cm × 10 cm, with three plants per hill in the field experiment. Each pot was transplanted into four hills with three plants per hill in pot experiment. The diameter of the pot was 35 cm and the height was 30 cm. The fertilization standard was composed of nitrogen (150 kg per ha as urea), phosphorus (100 kg per ha as diammonium phosphate), and potassium (75 kg per ha as potassium sulfate). Urea was also used at mid-tillering (100 kg per ha) as a top dressing.

### Experimental Design

A split-plot design was used for this experiment. Drought stress with different soil water at the jointing-booting stage was set as the whole-plot factor with three levels: mild drought stress (−10 kPa), moderate drought stress (−25 kPa), and severe drought stress (−40 kPa), and irrigation at 3 cm depth was used as a control (0 kPa). The water potential of SJ6 is denoted as A0 (0 kPa), A1 (−10 kPa), A2 (−25 kPa), and A3 (−40 kPa), and DN425 was marked as B0 (0 kPa), B1 (−10 kPa), B2 (−25 kPa), and B3 (−40 kPa). The booting stage was determined by manually dissecting the stem and visually observing it. The subplot factor was rice variety, and the area of the subplot was 35 m^2^. A large soil ridge was used to separate the plots (Supplementary Figure 1). The booting stage was defined as 50% of the plants having panicles that were visible to the naked eye as a tiny and transparent growth <2 mm in length buried within the leaf sheaths near the base of the plant, approximately 2 weeks before heading. The drought stress treatment lasted for 15 days. Irrigation was controlled before the drought stress to gradually reduce the soil water potential. A small amount of water was added to maintain the potential for the design if there was a water shortage during the comparable stress period. All treatments were placed under a rainproof shelter during drought stress. Eight soil tensiometers were evenly placed in two rows in each plot (Supplementary Figure 1). A soil tensiometer (Institute of Soil Science, Chinese Academy of Sciences) was used to monitor the soil water potential at a depth of 20 cm. The soil potential was monitored daily at 06:00, 12:00, and 18:00. When the soil water potential was low, an appropriate water supply was used to maintain the soil water potential within the range of ±3 kPa of the designed water potential. Regular irrigation was restored immediately after the drought stress. The soil water potential was rapidly restored to 0 kPa and the water depth was maintained at 3 cm until maturity. For the convenience of description, days after drought stress were recorded as Dx days, and days after restoration of irrigation were recorded as Rx days.

### Collection of Leaf Tissue for Physiological Determination and RNA-Sequencing

Three fully unfolded top leaves of each plant were collected and immediately flash frozen in liquid nitrogen in 2016. At each time point, leaves from 12 plants in the same treatment group were collected, and four plants were pooled for each biological replicate, resulting in three biological replicates per treatment group at each time point. The leaves were stored at −80°C until physiological determination and RNA extraction.

### Determination of Physiological Indicators

Sucrose content was measured using the resorcinol method (Du et al., 2020), with some modifications. 0.1 g leaves were ground with a mortar and pestle in liquid nitrogen and homogenized in 8 mL 80% ethanol, followed by an 80°C water bath for 40 min. Repeated extraction once and merged the extract. Activated carbon was added to the extracting solution, decolorized by an 80°C water bath for 30 min. The extracting solution was a crude enzyme solution with a constant volume of 20 mL. One hundred microliters of 2 mol⋅L^–1^ NaOH were added to a 4 mL crude enzyme solution, boiled in a water bath for 5 min. Seven milliliters of 30% hydrochloric acid and 2 mL 0.1% resorcinol were added successively, followed by an 80°C water bath for 10 min, and then the absorbance was measured at 480 nm.

The activity of VIN (S-AI) was determined as previously described (Yu et al., 2017), with some modifications. 0.1 g leaves were ground with a mortar and pestle in liquid nitrogen and homogenized in 1 mL extracting solution [50 mM HEPES-NaOH (pH 7.5), 50 mM MgCl_2_, 2 mM EDTA, 0.2% BSA, 2% PVP, 10 mM DTT]. The homogenate was centrifuged at 12,000 × *g* for 10 min at 4°C. The supernatant was a crude enzyme solution. Fifty microliters of crude enzyme solution were added to the determination and control tubes. Then, 200 μL 8% sucrose and 200 μL acetic acid-sodium acetate buffer solution was added to the determination and control tubes, respectively. Water baths were run at 37°C for 30 min, followed by a 95°C water bath for 10 min. One hundred twenty-five microliters of 20 mM dinitrosalicylic acid were added to the determination and control tubes, followed by a 95°C water bath for 10 min, and then the absorbance was measured at 510 nm. The catalyzed production of 1 μg reducing sugar per minute per gram leaf is defined as one unit of enzyme activity.

The activity of sucrose phosphate synthase (SPS) and sucrose synthase (SuSase) was determined as previously described (Yu et al., 2016), with some modifications. 0.5 g leaves were ground with a mortar and pestle in liquid nitrogen and homogenized in 3 mL extracting solution [50 mM HEPES-NaOH (pH 7.5), 50 mM MgCl_2_, 2 mM EDTA, 0.2% BSA, 2% PVP]. The homogenate was centrifuged at 10,000 × *g* for 10 min at 4°C. The supernatant was a crude enzyme solution. Fifty microliters of HEPES-NaOH, 20 μL 20 mM MgCl_2_, 20 μL 100 mM UDPG and 20 μL 100 mM fructose-6-phosphate (sucrose synthase determination with fructose added) were added to 50 μL crude enzyme solution, respectively. Then, water baths were run at 37°C for 30 min. Then, 200 μL 2 M NaOH was added to stop the reaction, boiled in a water bath for 10 min. One and a half milliliters of 30% hydrochloric acid and 0.5 mL 0.1% resorcinol were added successively, followed by an 80°C water bath for 10 min, and then the absorbance was measured at 480 nm. The catalyzed production of 1 μg sucrose per minute per gram leaf is defined as one unit of enzyme activity.

The activity of RuBisCO was determined as previously described (Wang et al., 1992), with some modifications. 0.1 g leaves were ground with a mortar and pestle in liquid nitrogen and homogenized in 1 mL extracting solution (1 M Tris–HCl buffer (pH 7.2), 20 mM KCl, 1 mM EDTA). Two hundred watt ultrasonic crushed 3 s, 7 s interval, a total time of 1 min. The homogenate was centrifuged at 8,000 × *g* for 10 min at 4°C. The supernatant was a crude enzyme solution. Ten microliters of 25 mM RuBP, 180 μL working solution (10 mM NaHCO_3_, 2.5 mM ATP, 0.3 mM NADH, 10 mM DTT, 5 mM MgCl_2_, 40 units⋅mL^–1^ 3-phosphoglycerate kinase, 40 units⋅mL^–1^ glyceraldehyde-3-phosphate dehydrogenase) were added to 10 μL crude enzyme solution, and then the absorbance was measured at 340 nm. The oxidation of 1 nmol NADH per minute per gram leaf is defined as one unit of enzyme activity.

The activity of FBA was determined as previously described (Schaeffer et al., 1997), with some modifications. 0.1 g leaves were ground with a mortar and pestle in liquid nitrogen and homogenized in 1 mL 50 mM Tris–HCl buffer (pH 7.5). Two hundred watt ultrasonic crushed 3 s, 7 s interval, a total time of 1 min. The homogenate was centrifuged at 8,000 × *g* for 10 min at 4°C. The supernatant was a crude enzyme solution. One hundred microliters of working solution (50 mM Tris–HCl, 5 mM MgCl_2_, 1 mM EDTA), 20 μL 210 μM NADH, 20 μL 2 mM fructose-1,6-bisphosphate, 20 μL 1 unit ml^–1^ glycerol-3-phosphate dehydrogenase and 20 μL 1 unit ml^–1^ triose-phosphate isomerase was added to 20 μL crude enzyme solution, and then the absorbance was measured at 340 nm. The consumption of 1 nmol NADH per minute per gram leaf is defined as one unit of enzyme activity.

### Weighted Gene Co-expression Network Analysis Network Analysis

Drought time datasets of the two cultivars were filtered to remove any genes that did not reach an FPKM value of 1 in at least one time point. Log2 normalized FPKM values were used to generate the co-expression network using cloud computing^1^. Average hierarchical clustering using the hclust function was performed to group the genes based on the topological overlap dissimilarity measure (1-TOM) of their connection strengths. Network modules were identified using a dynamic tree cut algorithm with a minimum cluster size of 30 and merging threshold function of 0.85. To relate the physiology measurements with the network, the module eigengenes were correlated with the physiological data. Correlations were performed for each physiological trait separately using the mean values at each time point to associate the diel patterns between the physiological traits and eigengenes. To associate individual genes with physiology, we calculated gene significance (GS) as described in the WGCNA package as the absolute value of the correlation between gene expression and physiology across the time series.

### qRT-PCR Confirmation of Differentially Expressed Genes

qRT-PCR was conducted using a LightCycler 480 real-time PCR system. Each reaction was performed in a volume of 20 μL containing 10 μL of 2 × SYBR green master reagent (TOYOBO, Japan), 2.0 μL of diluted transcription product and 0.6 μL of each gene-specific primer and 6.8 μL of ddH_2_O. The following thermal cycling conditions were used: 95°C for 5 min, followed by 45 cycles of 95°C for 15 s, 56°C for 25 s, and 72°C for 35 s. Dissociation curve analysis was performed using the following thermal profile: 95°C for 10 s, 60°C for 1 min, and 95°C for 15 min.

### Statistical Analysis

Data analyses were performed using the SPSS 18.0 (Chicago, IL, United States) software package. Analysis of variance (ANOVA) was used to analyze all data and differences among treatments. Results are reported as the mean ± standard deviation (SD) values of three independent experiments, by measuring at least three different replicates (plants) in each experiment. SD was calculated directly from crude data. The levels of significance in the figures are given by ns for not significant, * and ^**^ for significance at *P* < 0.05, and *P* < 0.01, respectively.

## Results

### Effects of Drought Stress on Photosynthesis

**FIGURE 2:**
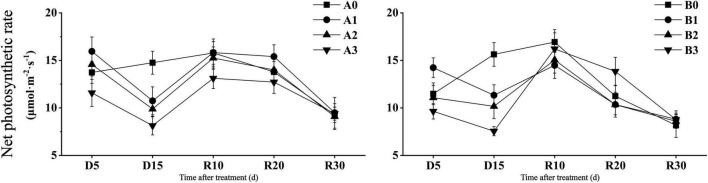
Effect of drought stress on photosynthetic parameters of growth period overlapping rice at jointing-booting stage. Vertical bars represent standard deviation. A0 and B0, control treatments (0 kPa) of SJ6 and DN425; A1 and B1, mild drought stress treatments (–10 kPa); A2 and B2, moderate drought stress treatments (–25 kPa); A3 and B3, severe drought stress (–40 kPa).

As shown in Figure 2, the net photosynthetic rate of the two cultivars decreased significantly in D15 days under drought stress. Compared with that of the control, the net photosynthetic rate of the two cultivars decreased by 27.24 and 27.51% under mild drought stress, 33.06 and 34.97% under moderate drought stress, and 44.89 and 51.70% under severe drought stress, respectively. After the restoration of irrigation, DN425 showed a stronger recovery ability than that of SJ6 under severe drought stress. Compared with D15, the net photosynthetic rate of the two cultivars under severe drought stress increased by 61.24 and 114.66% in R10 days, respectively. The net photosynthetic rate of DN425 under severe drought stress at R20 was significantly higher than that of the control.

**FIGURE 3:**
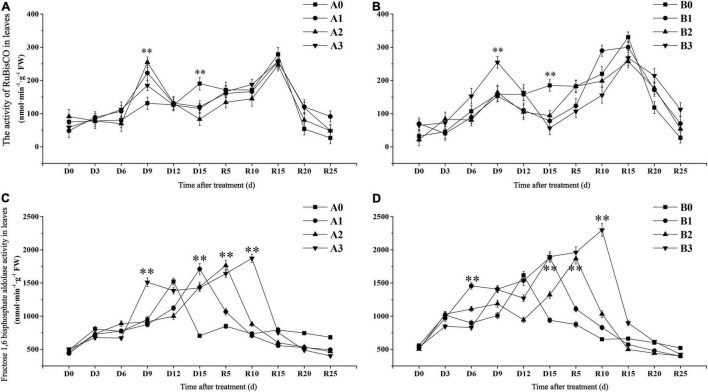
Effect of drought stress at jointing-booting stage on the activity of RuBisCO **(A,B)** and fructose-1,6-diphosphate aldolase **(C,D)** in growth period overlapping rice leaves. ^**^ represent significance at *P* < 0.01, respectively.

Ribulose-1,5-bisphosphate carboxylase/oxygenase is a key enzyme in the carbon fixation reaction of rice. The activity of RuBisCO in the control treatments reached the maximum value on day R15 and then decreased gradually (Figures 3A,B). In contrast to the control, the RuBisCO activity of the two cultivars under drought stress showed a bimodal curve change. The activity of RuBisCO in drought stress treatment reached its first peak on D9 days, which was significantly higher than that in the control. The activity of RuBisCO in drought stress treatments of SJ6 on D9 days was increased by 69.40, 93.88, and 40.82%, with increasing drought intensity, respectively. In contrast to SJ6, DN425 was significantly higher than the control only under severe drought stress on D9 days, and increased by 61.02% compared with the control. The activity of RuBisCO in drought stress treatments decreased rapidly after reaching the first peak, and was overall significantly lower than that in the control at the same time until D15 days. Compared with the control, the activity of RuBisCO in drought stress treatment of SJ6 decreased by 36.62, 56.34, and 39.44%, with increasing drought intensity, respectively. DN425 decreased by 57.97, 49.27, and 69.57%, respectively. After irrigation resumed, the activity of RuBisCO in each treatment decreased rapidly after reaching the second peak value on day R15. The RuBisCO activity of DN425 under moderate and severe drought stress did not reach that of the control after R15 days.

Fructose-1,6-bisphosphate aldolase (FBA) plays a key role in the regeneration of RuBP in plants and indirectly participates in plant photosynthesis. The maximum time of FBA enzyme activity was different under different conditions of drought stress (Figures 3C,D). The FBA activity of the two cultivars in the control treatment reached the maximum value on D12 days, and then decreased rapidly. FBA activity of the two cultivars under the mild, moderate and severe drought stress conditions peaked at D15, R5, and R10, respectively. The maximum activity of FBA in SJ6 treatment was 12.38, 15.89, and 23.03% higher than that in the control, with increasing drought intensity, respectively. DN425 increased by 16.27, 15.48, and 42.25%, respectively. These results indicate that drought stress increases the maximum activity of FBA and prolongs the time required to reach the maximum value. The more severe the drought stress, the greater the increase in the maximum activity of FBA and the longer the time required to reach the maximum value.

### Effects of Drought Stress on Sucrose Metabolism

#### Drought Stress Reduced Sucrose Content in Leaves

**FIGURE 4:**
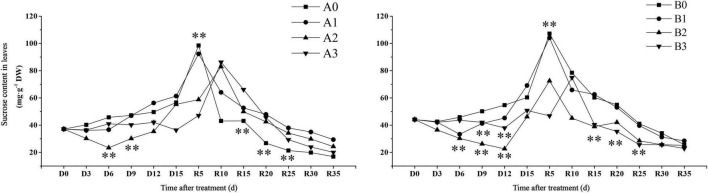
Effect of drought stress on sucrose content of growth period overlapping rice leaves at jointing-booting stage. ^**^ represent significance at *P* < 0.01, respectively.

Sucrose content is an important trait that reflects carbon sequestration in plant photosynthesis. The time when sucrose content reached maximum was found to be different for each treatment (Figure 4). The sucrose content in the leaves of the control treatment reached its maximum value on day R5. The sucrose content of SJ6 under moderate drought was significantly lower than that of the other treatments in the first 9 days after drought stress. The sucrose content of SJ6 under severe drought stress was significantly lower than that of the control, and the sucrose content was 35.77% lower than that of the control at D15 days. The sucrose content of DN425 under moderate and severe drought was significantly lower than that of the control, and decreased by 23.53 and 15.83% at D15, respectively. The sucrose content of mild drought stress was significantly higher than that of the control on D15 days, and increased by 14.50%. After the restoration of irrigation, the sucrose content under mild drought stress of the two cultivars reached the maximum value on R5 days, with no significant difference from the control, while the moderate and severe drought stress of the two cultivars reached the maximum value on R5 or R10 days, and the maximum value was significantly lower than that of the control. The sucrose content of SJ6 leaves under drought stress was significantly higher than that of the control from R10 to R25 days. The sucrose content of DN425 was significantly lower than that of the control from R15 to R25 days under moderate and severe drought stress. Drought stress also significantly affected the soluble sucrose content of leaves (Supplementary Figure 2).

#### Drought Stress Affects Sucrose Metabolism Pathway

##### Sucrose Synthase

**FIGURE 5:**
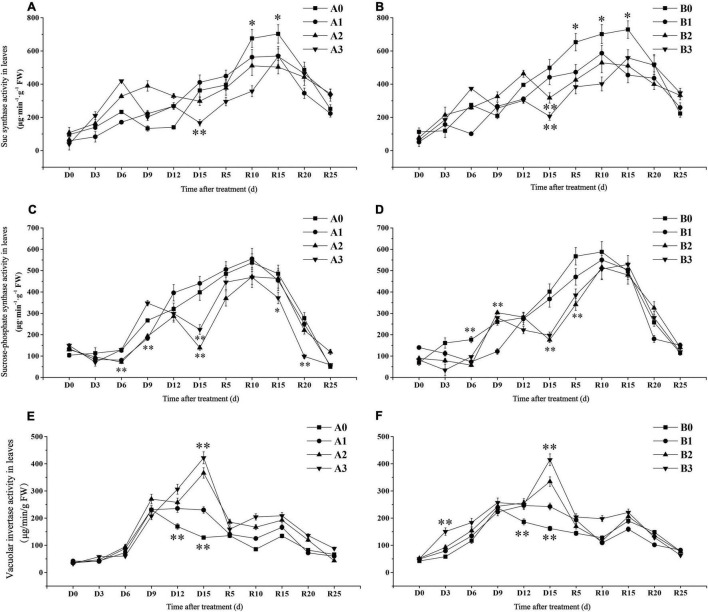
Effect of drought stress at jointing-booting stage on Sucrose synthase **(A,B)**, sucrose phosphate synthase **(C,D)** and vacuolar invertase **(E,F)** activity in leaves. *, ^**^ represent significance at *P* < 0.05 and *P* < 0.01, respectively.

The SuSase activity of SJ6 under moderate and severe drought stress was significantly higher than that of the control treatment on D6 days, while SuSase activity began to decline on D9 and D6 days, respectively (Figures 5A,B). SuSase activity under severe drought stress was 53.84% lower than that of the control on D15 days. SuSase activity of DN425 under moderate and severe drought stress began to decrease after D12 and D6, and was significantly lower than that in the control at D15 days, decreasing by 36.41 and 58.60%, respectively. After irrigation resumed, the maximum activity of SuSase decreased by 18.92, 27.34, and 19.37% in SJ6 and 19.59, 27.50, and 23.47% in DN425 with increasing drought intensity, compared with the control, respectively.

##### Sucrose Phosphate Synthase

Sucrose phosphate synthase activity of SJ6 under moderate drought stress decreased significantly after D12 and was 65.17% lower than that of the control at D15 (Figures 5C,D). Although the SPS activity of severe drought stress was temporarily higher than that of the control at D9 days, the SPS activity decreased again, which was 43.64% lower than that of the control at D15 days. SPS activity of DN425 increased rapidly under moderate and severe drought stress, reaching the control level on D9 days, and then decreasing again by 56.35 and 50.93%, respectively, at D15 days. After irrigation resumed, there was no significant difference in activity between the mild drought stress of SJ6 and that of the control. SPS activity under severe drought stress was significantly lower than that of the other treatments after R15 days. There was no significant difference in the maximum value of SPS activity of DN425 under the three different stress treatments.

##### Vacuolar Invertase

Vacuoles invertase activity continued to increase after D9 days under moderate and severe drought stress, and reached the maximum value on D15 days (Figures 5E,F). VIN activity of SJ6 under the three drought stress treatments was significantly higher than that of the control at D15 days, which increased by 78.93, 184.89, and 228.32%, with increasing drought intensity, respectively. DN425 was 49.90, 106.71, and 156.30% higher than that of the control, respectively. After the restoration of irrigation, the VIN activity of SJ6 under drought stress was significantly higher than that of the control, and gradually decreased to the control level after R15 days. The VIN activity of DN425 under severe drought stress was significantly higher than that of the control within 10 days of irrigation restoration.

#### Drought Stress Affects the Synthesis of Transient Starch in Leaves

**FIGURE 6:**
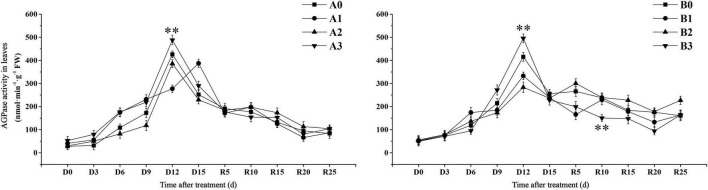
Effect of drought stress at jointing-booting stage on AGPase activity in growth period overlapping rice leaves. ** represent significance at *P* < 0.01, respectively.

We further examined the transient starch synthesis pathways in the leaves. The activity of APDG pyrophosphorylase (AGPase) in the control treatment reached the maximum value on D12 days (Figure 6). AGPase activity of SJ6 under mild and severe drought stress was significantly higher than that of the control after D6 days. AGPase activity under moderate and severe drought stress reached the maximum value on D12 days, AGPase activity under severe drought stress was 14.62% higher than that of the control. AGPase activity reached its maximum value on D15 days under mild drought stress. The AGPase activity of DN425 under mild and severe drought reached the maximum value on D12 days, while that of moderate drought reached the maximum value on day R5. AGPase activity increased by 19.32% on D12 days under severe drought, and decreased by 19.87 and 31.73% under mild and moderate drought, respectively.

#### Effects of Drought Stress on Rice Growth Process

**FIGURE 7:**
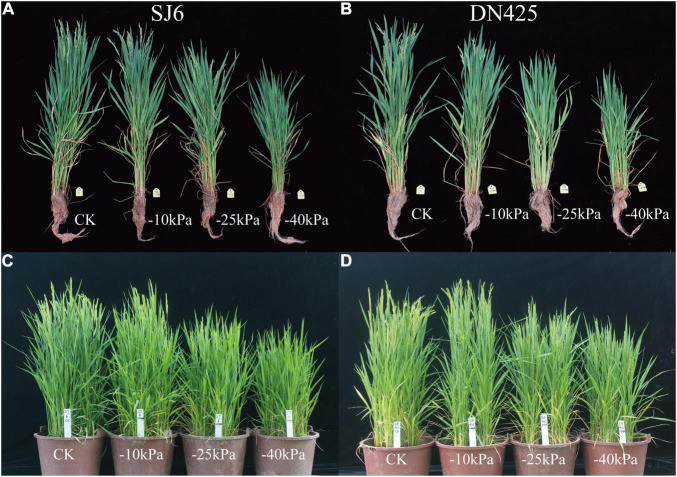
Effects of drought stress on rice growth process in field test **(A,B)** and pot experiment **(C,D)**.

In previous studies, drought stress was generally considered to shorten the vegetative growth period and advance the reproductive growth of rice. In our study on rice with overlapping growth periods, we found that mild and moderate drought stress accelerated the growth process of rice, and both the heading and flowering stages were occurred earlier (2–3 days) than the control (Figures 7A,B). However, severe drought stress caused a significant delay in the growth period. The two cultivars did not head after 15 days of drought stress, and the heading date was delayed by approximately 5 days compared with the control. To verify this result, pot cultivation was performed. The growth period of rice was generally earlier under pot conditions than under field conditions, but the growth period was also significantly delayed under severe drought stress (Figures 7C,D).

#### Differentially Expressed Genes of Carbon Metabolism in Rice With Overlapping Growth Periods Under Drought Stress at Jointing-Booting Stage

##### Transcriptome Sequencing Data Statistics

To better understand the transcriptome differences between the two cultivars under drought stress, transcriptome sequencing was performed on the leaves of SJ6 and DN425 during severe drought stress. Previous studies found that there were too many apoptotic genes on the 15th day after drought stress, which affected the analysis results. Therefore, only transcripts from 12 days before drought stress were selected for the analysis. Comparing the results with the reference genome showed that the comparison efficiency of reads of all samples with the reference genome ranged from 80.94 to 87.33% (Supplementary Table 1). About 37.79 million reads and 37.03 million reads in SJ6 and DN425, respectively, could be compared at least once to the reference genome.

#### Identification of Differentially Expressed Genes

##### Differential Expression Genes Have Time Effect Under Drought Stress

**FIGURE 8:**
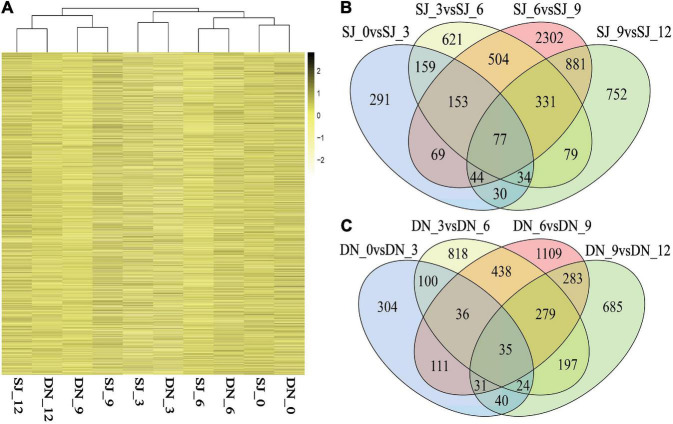
Analysis of transcription differences under drought stress. **(A)** Cluster analysis of differential expression genes. **(B)** Venn diagram of differential expression genes of SJ6 adjacent time samples. **(C)** Venn diagram of differential expression genes of DN425 adjacent time samples.

First, the gene whose expression level (FPKM) of all samples was not 0 was analyzed by clustering analysis (Figure 8A). The results showed that all samples of the two varieties were not clustered by variety difference, but by drought time difference, indicating that the effect of drought stress time on the growth period of overlapping rice was greater than that of inter-variety difference. Therefore, differentially expressed genes at different drought stress times were identified in the two cultivars.

A fold change of ≥2 and FDR of <0.01 were used as screening criteria in this study. The results showed that the number of differentially expressed genes (DEGs) was the highest from day 6 to day 9 under drought stress. The DEGs Venn diagram of SJ6 and DN425 at adjacent time nodes under drought stress (Figures 8B,C) showed that the number of DEGs unique to drought stress from 6 to 9 days was the largest, and 2,302 and 1,109 DEGs were identified, respectively, accounting for 52.79 and 47.76% of the total number of DEGs from 6 to 9 days, respectively. These results indicated that there were many unique differential genes expressed from the 6th to 9th day of drought stress, which were different from that of other time nodes.

#### Kyoto Encyclopedia of Genes and Genomes Enrichment Analysis Time-Specific Expression Genes

**FIGURE 9:**
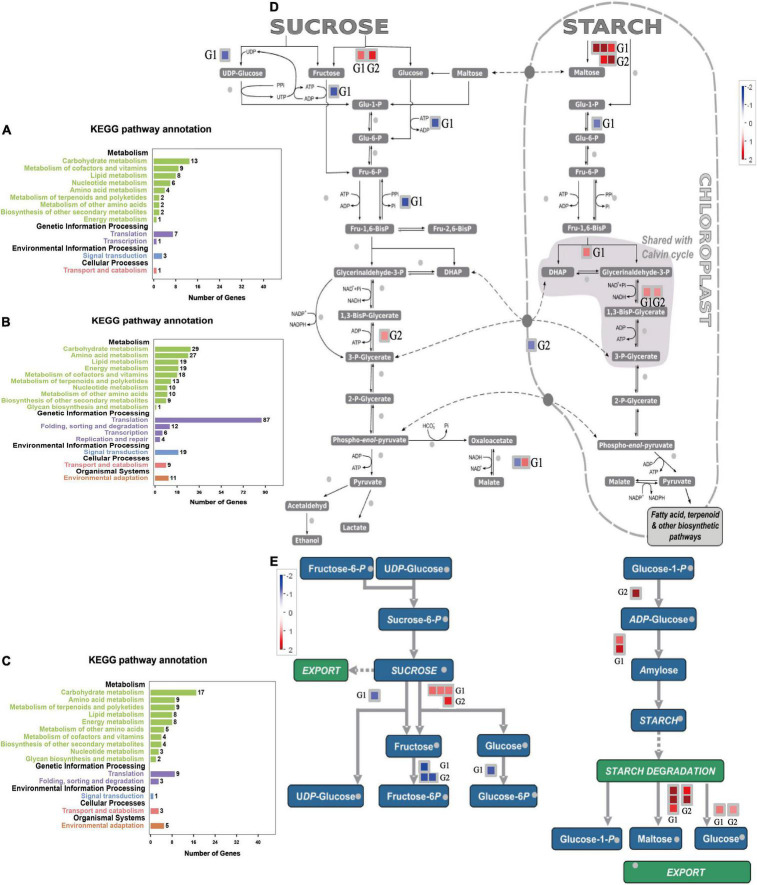
KEGG enrichment analysis of special DEGs from the 6th to the 9th day of severe drought stress. **(A)** KEGG enrichment analysis of time-specific DEGs of SJ6 and DN425 co-expressed at 6–9 days under severe drought stress. **(B)** KEGG enrichment analysis of SJ6 time-specific DEGs at 6–9 days under severe drought stress. **(C)** KEGG enrichment analysis of DN425 time-specific DEGs at 6–9 days under severe drought stress. **(D,E)** MapMan analysis of sucrose and starch metabolism of DEGs at 6–9 days under severe drought stress. G1, SJ6 time-specific DEGs at 6–9 days; G2, DN425 time-specific DEGs at 6–9 days.

We isolated the genes expressed at 6–9 days of drought stress from other time nodes to analyze the pathways of these DEGs. The main enriched pathways included carbohydrate metabolism, coenzyme and vitamin metabolism, and lipid metabolism (Figure 9A). Time-specific DEGs at 6–9 days of SJ6 were mainly enriched in translation, carbohydrate metabolism, and amino acid metabolism (Figure 9B). Time-specific DEGs at 6–9 days of DN425 were mainly enriched in carbohydrate metabolism, amino acid metabolism, terpenoids, and polyketone metabolism (Figure 9C).

According to the above results, during 6–9 days of severe drought stress, SJ6 and DN425 cultivars expressed more DEGs in the carbohydrate metabolism pathway, which were different from those of other time nodes, and there were differences among cultivars. Both cultivars expressed invertase genes during sucrose degradation (Figures 9D,E), SJ6 upregulated two invertase genes (LOC_Os03g20020 and LOC_Os09g08072), and DN425 upregulated only one invertase gene (LOC_Os03g20020). There may be an interaction between both invertases and multiple sucrose synthases (Supplementary Figures 3A,B). SJ6 was downregulated in UDP-glucose, fructose-6-phosphate, and glucose-6-phosphate pathways, indicating that sucrose degradation was not used for subsequent product synthesis. DN425 was also downregulated in the fructose-6-phosphate pathway. In starch synthesis, SJ6 upregulated the expression of two starch synthase genes, and DN425 upregulated the expression of ADP-glucose synthase-related enzyme genes. SJ6 upregulated three β-amylase genes (LOC_Os03g04770, LOC_Os10g32810, and LOC_Os03g22790) in starch degradation. DN425 upregulated the expression of two β-amylase genes (LOC_Os03g04770 and LOC_Os07g35880). Fructose-1,6-diphosphate metabolite related enzyme genes were up-regulated in both cultivars, and phosphate-transport-related genes were downregulated in DN425, whereas no downregulation was found in SJ6. Malate dehydrogenase related genes were also upregulated in SJ6.

#### Co-expression Network Analysis

To further explore the transcriptional changes in sucrose metabolism during the whole drought stress period, co-expression network analysis was performed on the two tested cultivars. The gene co-expression network was constructed, and gene modules were identified in the two cultivars under different drought stress conditions. The SJ6 expression genes constructed 12 modules, and DN425 expression genes constructed 13 modules (Supplementary Figure 4).

**FIGURE 10:**
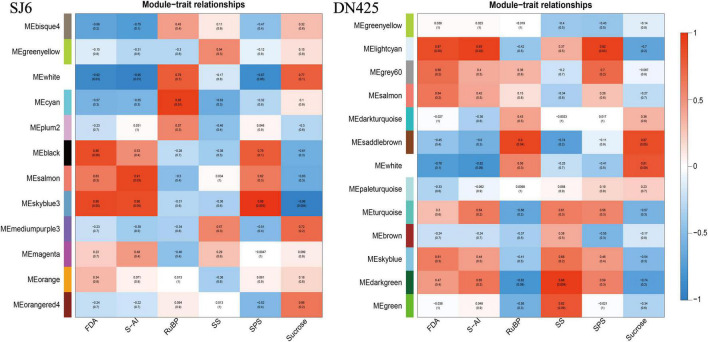
Correlations between module eigengenes and physiology measurements for SJ6 and DN425.

To correlate gene expression patterns with physiological trait data, weighted gene co-expression network analysis (WGCNA) was used to correlate different module genes with measured physiological traits (Figure 10). Six modules of SJ6 genes had the highest correlation with physiological traits (white, cyan, black, salmon, Skyblue3, and Mediumpurple3). Seven modules (lightcyan, Grey60, Saddlebrown, White, Turquoise, Skyblue, and Darkgreen) had the highest correlation with DN425 physiological traits.

#### Screening of Key Genes in Relevant Modules

**FIGURE 11:**
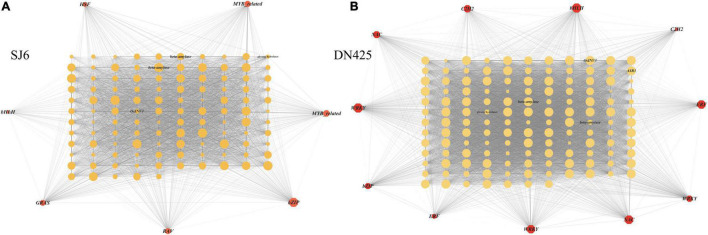
Gene co-expression network analysis shown by the Cytoscape. **(A)** Gene co-expression network of SJ6. **(B)** Gene co-expression network of DN425. The red polygon represents the transcription factors in the co-expression network, and the middle circle represents the co-expressed DEGs. The size of polygon and circle represents the degree of gene association, and the transparency of line between graphs represents the weight.

Co-expression network analysis of genes in the modules highly correlated with physiological traits in the above studies, revealing that a vacuolar invertase gene (LOC_Os02g01590) and its co-expression network were always upregulated during drought stress (Figure 11). It was found that the expression of this module gene was regulated by bZIP transcription factor (*OsbZIP04*) (Figure 11A). Among the highly correlated module genes of DN425, the WRKY transcription factor (*OsWRKY62*) showed the greatest degree of change, suggesting that it may play a key role in regulating gene expression (Figure 11B). A β-amylase gene and an ABA-stress-ripening inducible gene were also found in DN425 modules, and both genes were regulated by WRKY transcription factors. In addition, protein interaction prediction showed that the vacuolar invertase gene LOC_Os02g01590 interacted with multiple sucrose synthases and sucrose phosphate synthases (Supplementary Figure 3C).

#### Validation of Differentially Expressed Genes in RNA-Sequencing by Quantitative Reverse Transcription Polymerase Chain Reaction

To verify the accuracy of transcriptome sequencing, eight upregulated genes in the sequencing results were selected for real-time fluorescence quantitative PCR (qRT-PCR) validation (Supplementary Figure 5). The gene names and primer designs are listed in Supplementary Table 3. The qRT-PCR data were consistent with the RNA sequencing data, indicating the reliability of the RNA-Seq results.

## Discussion

### Effects of Drought Stress at Jointing-Booting Stage on Photosynthesis of Rice With Overlapping Growth Periods

Photosynthesis is the energy source for all the physiological activities of crops. Drought stress significantly reduces the photosynthetic capacity of rice and reduces photosynthetic products (Lu et al., 1994). RuBisCO and FBA are both key enzymes in the Calvin cycle, and their activities determine the photosynthetic carbon sequestration efficiency of rice. Some studies have found that because RuBisCO is a bifunctional enzyme, its activity is affected by CO_2_ concentration regulation, drought stress reduces mesophyll conductance, causing abnormal CO_2_ transmission (Ding et al., 2014), and resulting in an increased photorespiration rate and a decrease in net photosynthetic rate (Kozaki and Takeba, 1996; Wingler et al., 2000; Guo et al., 2008). Some scholars believe that severe water stress can lead to a dual decline in RuBisCO activity and content (Chen et al., 1999; Perdomo et al., 2017). However, some studies have found that the expression of RuBisCO activase is upregulated under drought stress, thus increasing the activity of RuBisCO (Yue et al., 2015; Perdomo et al., 2017). In this study, the activity of RuBisCO increased rapidly after the onset of drought stress and reached its first peak at D9. The activity of RuBisCO under drought stress was significantly higher than that of the control, indicating that short-term drought stress promoted the activity of RuBisCO. We believe that leaf water loss and chloroplast degradation continued under drought stress, leading to a decrease in CO_2_ transport capacity and photosynthetic rate. A large amount of RuBisCO was activated to compensate for the decreased photosynthetic carbon sequestration capacity caused by chloroplast degradation. However, with further water loss caused by drought stress, the mesophyll conductance decreased, and CO_2_ transport capacity was significantly reduced, which greatly reduced the carboxylation capacity (Ding et al., 2014). This explains why RuBisCO activity decreased rapidly after reaching its first peak during drought stress.

Fructose-1,6-bisphosphate aldolase is also a key enzyme in photosynthesis (Toshio et al., 1991), and both gene expression and enzyme activity have, under different circumstances, been shown to increase in a variety of stress-related studies (Yamada et al., 2000; Purev et al., 2008; Yang, 2009; Liu et al., 2019). FBA is generally believed to have four main functions in cells: participation in osmotic regulation (Fan, 2009), increased photosynthetic rate under stress (Long et al., 2010), participation in Na^+^/H^+^ transport (Yamada et al., 2000), and sucrose signaling (Lu, 2011). In this study, drought stress increased the maximum FBA activity and extended the time required to reach the maximum value. The more severe the degree of drought stress, the greater the maximum value of FBA activity and the longer the time required to reach the maximum value. In the drought stress stage, FBA activity increased with an increase in RuBisCO activity. It did not decrease with the decrease in RuBisCO activity but continued to increase until after the resumption of irrigation, before rapidly decreasing. Considering the diversity of FBA’s participation in biological processes, we believe that FBA, in the form of its reaction product triose phosphate, acts as a link during drought stress and after restoration of irrigation, that is, coordinating various metabolic pathways to jointly resist drought stress and complete post-stress recovery. During drought stress, FBA mainly participates in the regeneration reaction of RuBP, improving the efficiency of photosynthetic carbon fixation. When the activity of RuBisCO decreases rapidly due to drought stress, FBA activity continues to increase, producing more triose phosphate. On the one hand, RuBP regeneration is maintained. On the other hand, the subsequent reaction of triose phosphate ultimately provides raw materials for the production of hormones and other substances. Transcriptomic sequencing results showed that FBA-related enzyme genes and genes related to subsequent metabolic pathways were upregulated to varying degrees, confirming the above views. The FBA activity continued to increase after the restoration of irrigation, which may be due to the fact that the recovery process of drought stress requires a large amount of triose phosphate to synthesize the required substances, and the more severe the drought stress is, the longer the recovery time will be. The control treatment does not require such a recovery process, so the FBA activity does not need to be maintained at high levels. This also explains why the FBA activity of the control treatment remained stable at later stages of the experiment.

### Effects of Drought Stress on Sucrose Metabolism

Sucrose synthase, sucrose phosphate synthase, and acid invertase are the key enzymes involved in sucrose metabolism (Housley et al., 1989; Pollock and Cairns, 1991). Relevant studies have shown that the activities of SuSase and SPS are significantly increased under dehydration and osmotic stress (Yang et al., 2001, 2003; Romer et al., 2004; Klotz and Haagenson, 2008). Invertase is considered to be a key enzyme in cell osmotic regulation, which can hydrolyze sucrose into two hexose molecules, thus doubling osmotic pressure (Thomas and González, 2004; Sergeeva et al., 2006). Invertase has also been found to be involved in fruit formation in other crops (Zhang et al., 2006; Hayes et al., 2007). It was found that water stress increased SPS activity, but decreased invertase activity (Cao et al., 2018). In the study of abiotic stress in rice, some scholars found that the activities of SuSase and SPS were significantly increased in the hypertonic environment induced by salt stress (Shao et al., 2022). In this study, under moderate and severe drought stress, the activity of SuSase and SPS in leaves decreased after a period of stress, indicating that the sucrose synthesis and decomposition ability of leaves decreased at this time. However, VIN activity in leaves was significantly higher than that in the control at this stage, making up for low sucrose decomposition ability. Transcriptome sequencing also showed that the VIN gene was highly expressed at this time, and a variety of highly expressed invertase genes were enriched at the same time. Coupled with sucrose transportation, the sucrose content in leaves under moderate and severe drought stress increased slowly. Path analysis showed that the decrease in net photosynthetic rate was the main factor limiting sucrose content under severe drought stress (Supplementary Table 2). After restoration of irrigation, the maximum SPS activity of drought stress treatments was not significantly lower than that of the control, and the maximum SuSase activity was significantly lower than that of the control, while the VIN activity was significantly lower than that during drought stress, indicating that the sucrose synthesis ability of drought stress treatments was nearly at control levels, and sucrose decomposition capacity decreased significantly compared to that of the control. This also explains why the sucrose content of SJ6 leaves under drought stress was higher than that of the control in the recovery stage, studies have found similar results (Cao et al., 2018). In addition, in DN425, moderate and severe drought stress treatments recovered the sucrose content of leaves to a level significantly lower than that of the control, and panicle sucrose content was significantly higher than that of the control at this stage (Supplementary Figure 6); thus, we speculated that this phenomenon was due to sucrose transportation from leaves to grains. Related research has found that drought stress also increases the material transport phenomena (Wang et al., 2004). Path analysis showed that there was a complex correlation among VIN, SuSase, and SPS activities under different drought stress conditions, and the three enzymes jointly determined the sucrose content of leaves. The interaction prediction of the two VIN genes enriched in the transcriptome sequencing also revealed the interaction between the vacuolar invertase genes and a variety of sucrose synthases and sucrose phosphate synthases, which also confirmed the hypotheses above. The internal mechanism of sucrose content, determined by the interaction of the three enzymes, needs to be further studied.

### Effects of Drought Stress on Starch Metabolism

Scholars found that drought stress reduced the synthesis of transitory starch in leaves and enhanced the storage starch in developing grains (Prathap and Tyagi, 2020). The AGPase activity was found to be increased under water deficit stress in rice leaves (Prathap et al., 2019). Studies on drought stress in the rice booting stage have shown that it will have a great impact on rice (Guo et al., 2014; Chen et al., 2016; Zhao et al., 2017). In this study, AGPase activity of SJ6 leaves increased under drought stress, which was consistent with the results of Yang Jianchang’s study (Yang et al., 2003). AGPase activity decreased rapidly after D12 days, and decreased slowly after R5 days. These results indicate that after grain grouting began, the synthesis rate of instantaneous starch in the leaves decreased significantly. SuSase activity in leaves continued to increase after R5 days, which may be because not all sucrose degraded by SuSase was used in the synthesis of instantaneous starch because recovery after drought stress requires a material basis. Transcriptome analysis showed that genes related to UDP-glucose, fructose-6-phosphate, and glucose-6-phosphate synthesis were downregulated in SJ6 during drought stress, which also confirmed the hypotheses above. In addition, transcriptome sequencing showed that multiple upregulated β-amylase genes were enriched during drought stress, suggesting that starch was mainly metabolized by degradation rather than synthesis during drought stress.

### Effects of Drought Stress on Growth Process of Rice With Overlapping Growth Periods

Previous studies have suggested that drought stress can advance the reproductive growth of rice and accelerate the process of premature senescence. However, our study found that only mild and moderate drought stress could advance the growth period of rice with overlapping growth periods. When the soil water potential reached −40 kPa at the jointing-booting stage, that is, when severe drought stress was reached, the normal growth process of rice was interrupted. After irrigation was restored, the interrupted growth process under severe drought stress was resumed, which resulted in heading and anthesis occurring significantly later than those in the control. Transcriptome sequencing revealed that a delayed flowering gene (*OsABF1*) was upregulated during drought stress, and the same phenomenon was found in related studies (Zhang et al., 2016). The upregulated expression of *OsABF1* was not observed under mild and moderate drought stress.

### Weighted Gene Co-expression Network Analysis Key Gene Screening

Because time has a more significant effect on the transcriptome than changes in drought stress on the transcriptome, the difference at the transcriptome level in a single comparison of time cannot reflect the effect of drought stress at the gene level. Co-expression network analysis is an analytical method that leads to significant differences in treatment, development, or time scale by providing path structures (Priest et al., 2014; McClung et al., 2015; Righetti et al., 2016). In addition, co-expression network analysis can integrate transcriptome data into multiple datasets, which can be used for subsequent analysis of gene network structure and prediction of key genes within the structure (Langfelder et al., 2011; Krouk et al., 2013). In this study, we used weighted gene co-expression network analysis (WGCNA) (Langfelder and Horvath, 2008, 2012) to synthesize and analyze transcriptome and physiological data from 12 days of drought stress in both cultivars. Transcriptome data were classified based on the gene expression patterns during drought stress. The results showed that 12 and 13 gene modules were constructed for SJ6 and DN425, respectively, and six and seven gene modules were selected, which were highly positively correlated with physiological traits, respectively. Analysis of the gene expression network in the SJ6 module showed that the bZIP transcription factor (*OsbZIP04*) played a regulatory role in gene expression in most modules, especially in modules highly related to FBA and VIN enzymes. The bZIP transcription factor was found to be associated with most of the genes in the modules. Among them, the vacuolar invertase gene (LOC_Os02g01590) was upregulated during drought stress. Previous studies have also shown that the bZIP transcription factor plays a key role in drought resistance of rice (Fukao and Xiong, 2013; Maruyama et al., 2014); and in the analysis of the gene expression network of the LOC_Os02g01590 module in DN425, the WRKY transcription factor (*OsWRKY62*) is highly correlated with genes in the module. In contrast, the bZIP transcription factor is not highly correlated, and the β-amylase gene in the module is also found to be regulated by WRKY transcription factors. WRKY transcription factors have also been shown to be involved in abiotic stress resistance in rice genes (Song et al., 2010). These results indicate that SJ6 and DN425 differ in the regulatory pathways of related genes, but it may be that the two transcription factors jointly regulate the expression of related genes, but there is a strong and weak difference in the correlation. These predictions need to be verified in subsequent studies. In addition, in the analysis of gene networks in modules highly related to physiological traits, we also found a large number of other transcription factor families related to drought stress reported by predecessors, such as the AP2/EREBP transcription factor family, the zinc finger (Selvaraj et al., 2020), MYB (El-Kereamy et al., 2012) and the NAC transcription factors (Lee et al., 2017).

## Conclusion

Drought stress at the jointing-booting stage significantly reduced the net photosynthetic rate of rice leaves with overlapping growth stages during stress, and the more severe the drought stress, the greater the decrease. Drought stress promoted the activity of RuBisCO in a short time, but the activity of RuBisCO decreased after a period of drought. Drought stress increased the maximum activity of FBA, the more severe the drought stress, the greater the increase in the maximum activity of FBA and the longer the time required to reach the maximum value. The VIN activity of leaves increased significantly under drought stress, and the more severe the drought stress, the greater the increase. Drought stress significantly reduced the activities of SuSase and SPS in functional leaves and affected sucrose synthesis and degradation. After irrigation resumed, all treatments failed to recover to the control level. Severe drought stress changes the inherent growth process and delays the heading and flowering stages of rice. The number of DEGs was the highest from day 6 to day 9 under drought stress. Kyoto encyclopedia of genes and genomes (KEGG) enrichment analysis revealed that two invertase genes and four β-amylase genes with time-specific expression were involved in sucrose-starch metabolism in rice under drought stress. Combined with WGCNA analysis, VIN and β-amylase genes were regulated by *OsbZIP04* and *OsWRKY62* transcription factors during drought stress. Finally, our findings provide valuable insights into carbon fixation and sucrose metabolism in rice with overlapping growth periods under drought stress.

## Data Availability Statement

The datasets presented in this study can be found in online repositories. The names of the repository/repositories and accession number(s) can be found below: https://www.ncbi.nlm.nih.gov/, PRJNA815498.

## Author Contributions

XW: writing original draft, investigation, funding acquisition, and methodology. HL, DZ, ZQ, and BS: investigation and methodology. YJ, JW, and HLZ: project administration. DTZ and HWZ: review and editing, supervision, and funding acquisition. All authors contributed to the article and approved the submitted version.

## Conflict of Interest

The authors declare that the research was conducted in the absence of any commercial or financial relationships that could be construed as a potential conflict of interest.

## Publisher’s Note

All claims expressed in this article are solely those of the authors and do not necessarily represent those of their affiliated organizations, or those of the publisher, the editors and the reviewers. Any product that may be evaluated in this article, or claim that may be made by its manufacturer, is not guaranteed or endorsed by the publisher.

## Footnotes

1
https://console.biocloud.net

## Supplementary Material

Supplementary Figure 1 Field map of the experimental design. The red dotted line represents the separation between repetitions, and the black dotted line represents the partition between varieties. The circle represents the position of the soil tensiometer. The black line represents the soil ridge for separating treatments.

Supplementary Figure 2 Effect of drought stress on the soluble sugar content of growth period overlapping rice leaves at the jointing-booting stage. **Represent significance at *P* < 0.01, respectively.

Supplementary Figure 3 Prediction of invertase protein interaction network. **(A)** LOC_Os03g20020, **(B)** LOC_Os09g08072, and **(C)** LOC_Os02g01590.

Supplementary Figure 4 Gene merging module cluster tree.

Supplementary Figure 5 DEGs between RNA-Seq and qRT-PCR.

Supplementary Figure 6 Effect of drought stress on sucrose content of growth period overlapping rice panicles at the jointing-booting stage.

## References

[B1] Amthor J. McCree K. J. (1990). “Carbon balance of stressed plants: a conceptual model for integrating research results,” in *Stress Responses in Plants: Adaptation and Acclimation Mechanisms*, eds Cumming J. R. Alscher R. G. (Hoboken, NJ: Wiley), 1–15.

[B2] Baerenfaller K. Massonnet C. Walsh S. Baginsky S. Bühlmann P. Hennig L. (2012). Systems-based analysis of *Arabidopsis* leaf growth reveals adaptation to water deficit. *Mol. Syst. Biol.* 8:606. 10.1038/msb.2012.39 22929616 PMC3435506

[B3] Bolouri-Moghaddam M. R. Le Roy K. Xiang L. Rolland F. Van Den Ende W. (2010). Sugar signalling and antioxidant network connections in plant cells. *FEBS J.* 277 2022–2037. 10.1111/j.1742-4658.2010.07633.x 20412056

[B4] Caldeira C. F. Jeanguenin L. Chaumont F. Tardieu F. (2014). Circadian rhythms of hydraulic conductance and growth are enhanced by drought and improve plant performance. *Nat. Commun.* 5:5365. 10.1038/ncomms6365 25370944 PMC4241992

[B5] Cao X. C. Zhu C. Q. Zhong C. Hussain S. Zhu L. F. Wu L. H. (2018). Mixed-nitrogen nutrition-mediated enhancement of drought tolerance of rice seedlings associated with photosynthesis, hormone balance and carbohydrate partitioning. *Plant Growth Regul.* 84 451–465.

[B6] Chaitanya K. V. Jutur P. P. Sundar D. Ramachandra Reddy A. (2003). Water stress effects on photosynthesis in different mulberry cultivars. *Plant Growth Regul.* 40 75–80.

[B7] Chaves M. M. Flexas J. Pinheiro C. (2009). Photosynthesis under drought and salt stress: regulation mechanisms from whole plant to cell. *Ann. Bot.* 103 551–560. 10.1093/aob/mcn125 18662937 PMC2707345

[B8] Chen L. Wang B. Jiang Y. Cao C. Li P. (2016). Effects of drought and rewatering on rice physiological and biochemical indexes of leaves and grain yield at booting stage. *China Rice* 22 59–64.

[B9] Chen W. Zhao G. Gu Y. (1999). Advance of ribulose 1, 5 bisphosphate carboxylase/oxygenase (RubisCO). *Prog. Biochem. Biophys.* 26 433–436.

[B10] Couée I. Sulmon C. Gouesbet G. El Amrani A. (2006). Involvement of soluble sugars in reactive oxygen species balance and responses to oxidative stress in plants. *J. Exp. Bot.* 57 449–459. 10.1093/jxb/erj027 16397003

[B11] Ding L. Li Y. Li Y. Shen Q. Guo S. (2014). Effects of drought stress on photosynthesis and water status of rice leaves. *Chin. J. Rice Sci.* 28 65–70.

[B12] Du X. Zhang X. Xi M. Kong L. (2020). Split application enhances sweetpotato starch production by regulating the conversion of sucrose to starch under reduced nitrogen supply. *Plant Physiol. Biochem.* 151 743–750. 10.1016/j.plaphy.2020.04.027 32361224

[B13] Dubois M. Claeys H. Van Den Broeck L. Inzé D. (2017). Time of day determines *Arabidopsis* transcriptome and growth dynamics under mild drought. *Plant Cell Environ.* 40 180–189. 10.1111/pce.12809 27479938

[B14] El-Kereamy A. Bi Y. M. Ranathunge K. Beatty P. H. Good A. G. Rothstein S. J. (2012). The rice R2R3-MYB transcription factor OsMYB55 is involved in the tolerance to high temperature and modulates amino acid metabolism. *PLoS One* 7:e52030. 10.1371/journal.pone.0052030 23251677 PMC3522645

[B15] Fan W. (2009). *Cloning and Analysis of Salt-Induced Genes from Sesuvium portulacastrum Roots.* Haikou: Hainan University.

[B16] Fukao T. Xiong L. (2013). Genetic mechanisms conferring adaptation to submergence and drought in rice: simple or complex? *Curr. Opin. Plant Biol.* 16 196–204. 10.1016/j.pbi.2013.02.003 23453780

[B17] Gehan M. A. Greenham K. Mockler T. C. Mcclung C. R. (2015). Transcriptional networks-crops, clocks, and abiotic stress. *Curr. Opin. Plant Biol.* 24 39–46. 10.1016/j.pbi.2015.01.004 25646668

[B18] Greenham K. Guadagno C. R. Gehan M. A. Mockler T. C. Weinig C. Ewers B. E. (2017). Temporal network analysis identifies early physiological and transcriptomic indicators of mild drought in *Brassica rapa*. *eLife* 6:e29655. 10.7554/eLife.29655 28826479 PMC5628015

[B19] Greenham K. McClung C. R. (2015). Integrating circadian dynamics with physiological processes in plants. *Nat. Rev. Genet.* 16 598–610. 10.1038/nrg3976 26370901

[B20] Guo G. Liu H. Li G. Liu M. Li Y. Wang S. (2014). Analysis of physiological characteristics about ABA alleviating rice booting stage drought stress. *Sci. Agric. Sin.* 47 4380–4391.

[B21] Guo S. Zhou Y. Shen Q. Zhang F. (2008). Effect of ammonium and nitrate nutrition on some physiological processes in higher plants - growth, photosynthesis, photorespiration, and water relations. *Plant Biol.* 9 21–29. 10.1055/s-2006-924541 17048140

[B22] Hamilton J. G. Zangerl A. R. Delucia E. H. Berenbaum M. R. (2001). The carbon–nutrient balance hypothesis: its rise and fall. *Ecol. Lett.* 4 86–95.

[B23] Hayes M. A. Christopher D. Dry I. B. (2007). Isolation, functional characterization, and expression analysis of grapevine (*Vitis vinifera* L.) hexose transporters: differential roles in sink and source tissues. *J. Exp. Bot.* 58 1985–1997. 10.1093/jxb/erm061 17452752

[B24] Housley T. L. Kanabus J. Carpita N. C. (1989). Fructan synthesis in wheat leaf blades. *J. Plant Physiol.* 134 192–195.

[B25] Klotz K. L. Haagenson D. M. (2008). Wounding, anoxia and cold induce sugarbeet sucrose synthase transcriptional changes that are unrelated to protein expression and activity. *J. Plant Physiol.* 165 423–434. 10.1016/j.jplph.2007.02.001 17395334

[B26] Kozaki A. Takeba G. (1996). Photorespiration protects C3 plants from photooxidation. *Nature* 384 557–560.

[B27] Kozlowski T. T. Pallardy S. G. (2002). Acclimation and adaptive responses of woody plants to environmental stresses. *Bot. Rev.* 68 270–334.

[B28] Krouk G. Lingeman J. Colon A. Coruzzi G. Shasha D. (2013). Gene regulatory networks in plants: learning causality from time and perturbation. *Genome Biol.* 14:123. 10.1186/gb-2013-14-6-123 23805876 PMC3707030

[B29] Langfelder P. Horvath S. (2008). WGCNA: an R package for weighted correlation network analysis. *BMC Bioinformatics* 9:559. 10.1186/1471-2105-9-559 19114008 PMC2631488

[B30] Langfelder P. Horvath S. (2012). Fast R functions for robust correlations and hierarchical clustering. *J. Stat. Softw.* 46:i11. 23050260 PMC3465711

[B31] Langfelder P. Luo R. Oldham M. C. Horvath S. Bourne P. E. (2011). Is my network module preserved and reproducible? *PLoS Comput. Biol.* 7:e1001057. 10.1371/journal.pcbi.1001057 21283776 PMC3024255

[B32] Lee D. K. Chung P. J. Jeong J. S. Jang G. Bang S. W. Jung H. (2017). The rice OsNAC6 transcription factor orchestrates multiple molecular mechanisms involving root structural adaptions and nicotianamine biosynthesis for drought tolerance. *Plant Biotechnol. J.* 15 754–764. 10.1111/pbi.12673 27892643 PMC5425393

[B33] Liu C. Y. Zhu P. P. Fan W. Feng Y. Kou M. Hu J. (2019). Functional analysis of drought and salt tolerance mechanisms of mulberry RACK1 gene. *Tree Physiol.* 39 2055–2069. 10.1093/treephys/tpz108 31728533

[B34] Long R. Yang Q. Kang J. Wang P. Qin Z. (2010). Cloning and characterization of a frutose-1,6-bisphosphate aldolase gene in *Medicago sativa* L. *Acta Bot. Boreali Occidentalia Sin.* 30 1075–1082.

[B35] Lu C. Zhang Q. Kuang T. Wang Z. Gao Y. (1994). The mechanism for the inhibition of photosynthesis in rice by water stress. *Acta Agron. Sin.* 20 601–606.

[B36] Lu W. (2011). *Genome-Wide Analysis of the Fructose Bisphosphate Aldolases in Arabidosis.* Tai’an: Shandong Agricultural university.

[B37] Lv S. Zhang K. Gao Q. Lian L. Song Y. Zhang J. (2008). Overexpression of an H+-PPase gene from *Thellungiella halophila* in cotton enhances salt tolerance and improves growth and photosynthetic performance. *Plant Cell Physiol.* 49 1150–1164. 10.1093/pcp/pcn090 18550626

[B38] Martínez-Noël G. M. Tognetti J. A. Salerno G. L. Wiemken A. Pontis H. G. (2009). Protein phosphatase activity and sucrose-mediated induction of fructan synthesis in wheat. *Planta* 230 1071–1079. 10.1007/s00425-009-1002-7 19714360

[B39] Maruyama K. Urano K. Yoshiwara K. Morishita Y. Sakurai N. Suzuki H. (2014). Integrated analysis of the effects of cold and dehydration on rice metabolites, phytohormones, and gene transcripts. *Plant Physiol.* 164 1759–1771. 10.1104/pp.113.231720 24515831 PMC3982739

[B40] McClung C. Robertson G. Malia A. Greenham K. Mockler T. (2015). Transcriptional networks – crops, clocks, and abiotic stress. *Curr. Opin. Plant Biol.* 24 39–46. 25646668 10.1016/j.pbi.2015.01.004

[B41] National Bureau of Statistics of China [NBSC] (2017). *China Statistical Yearbook.* Beijing: National Bureau of Statistics of China.

[B42] Niedzwiedz-Siegien I. Bogatek-Leszczynska R. Côme D. Corbineau F. (2004). Effects of drying rate on dehydration sensitivity of excised wheat seedling shoots as related to sucrose metabolism and antioxidant enzyme activities. *Plant Sci.* 167 879–888.

[B43] Perdomo J. A. Sebastià C.-B. Elizabete C.-S. Jeroni G. (2017). Rubisco and rubisco activase play an important role in the biochemical limitations of photosynthesis in rice, wheat, and maize under high temperature and water deficit. *Front. Plant Sci.* 8:490. 10.3389/fpls.2017.00490 28450871 PMC5390490

[B44] Pollock C. J. Cairns A. J. (1991). Fructan metabolism in grasses and cereals. *Annu. Rev. Plant Physiol. Plant Mol. Biol.* 42 77–101.

[B45] Prathap V. Ali K. Singh A. Vishwakarma C. Krishnan V. Chinnusamy V. (2019). Starch accumulation in rice grains subjected to drought during grain filling stage. *Plant Physiol. Biochem.* 142 440–451. 10.1016/j.plaphy.2019.07.027 31419646

[B46] Prathap V. Tyagi A. (2020). Correlation between expression and activity of ADP glucose pyrophosphorylase and starch synthase and their role in starch accumulation during grain filling under drought stress in rice. *Plant Physiol. Biochem.* 157 239–243. 10.1016/j.plaphy.2020.10.018 33130401

[B47] Priest H. D. Fox S. E. Rowley E. R. Murray J. R. Michael T. P. Mockler T. C. (2014). Analysis of global gene expression in brachypodium distachyon reveals extensive network plasticity in response to abiotic stress. *PLoS One* 9:e87499. 10.1371/journal.pone.0087499 24489928 PMC3906199

[B48] Purev M. Kim M. K. Samdan N. Yang D. C. (2008). Isolation of a novel fructose-1,6-bisphosphate aldolase gene from *Codonopsis lanceolata* and analysis of the response of this gene to abiotic stresses. *Mol. Biol.* 42:179. 18610828

[B49] Righetti K. Vu J. Pelletier S. Vu B. L. Glaab E. Lalanne D. (2016). Inference of longevity-related genes from a robust coexpression network of seed maturation identifies regulators linking seed storability to biotic defense-related pathways. *Plant Cell* 27 2692–2708. 26410298 10.1105/tpc.15.00632 PMC4682330

[B50] Righetti K. Vu J. L. Pelletier S. Vu B. L. Glaab E. Lalanne D. (2015). Inference of longevity-related genes from a robust coexpression network of seed maturation identifies regulators linking seed storability to biotic defense-related pathways. *Plant Cell* 27 2692–2708. 10.1105/tpc.15.00632 26410298 PMC4682330

[B51] Romer U. Schrader H. Günther N. Nettelstroth N. Frommer W. B. Elling L. (2004). Expression, purification and characterization of recombinant sucrose synthase 1 from *Solanum tuberosum* L. for carbohydrate engineering. *J. Biotechnol.* 107 135–149. 10.1016/j.jbiotec.2003.10.017 14711497

[B52] Schaeffer G. W. Sharpe F. T. Sicher R. C. (1997). Fructose 1,6-bisphosphate aldolase activity in leaves of a rice mutant selected for enhanced lysine. *Phytochemistry* 46 1335–1338. 10.1016/s0031-9422(97)00470-6 9419899

[B53] Selvaraj M. Jan A. Ishizaki T. Valencia M. Dedicova B. Maruyama K. (2020). Expression of the CCCH-tandem zinc finger protein gene OsTZF5 under a stress-inducible promoter mitigates the effect of drought stress on rice grain yield under field conditions. *Plant Biotechnol. J.* 10 1111. 10.1111/pbi.13334 31930666 PMC7336284

[B54] Sergeeva L. Keurentjes J. Bentsink L. Vonk J. Van Der Plas L. Koornneef M. (2006). Vacuolar invertase regulates elongation of *Arabidopsis thaliana* roots as revealed by QTL and mutant analysis. *Proc. Natl. Acad. Sci. U.S.A.* 103 2994–2999. 10.1073/pnas.0511015103 16481625 PMC1413815

[B55] Shao X. W. Gai D. S. Gao D. P. Geng Y. Q. Guo L. Y. (2022). Effects of salt-alkaline stress on carbohydrate metabolism in rice seedlings. *Phyton Int. J. Exp. Bot.* 91 745–759.

[B56] Singh A. K. Choudhury B. U. Bouman B. A. M. (2002). “Water-wise rice production,” in *Proceedings of the The International Workshop on Water-Wise Rice Production*, eds Bouman B. A. M. Hengsdijk H. Hardy B. Bindraban B. Toung T. P. Ladha J. K. (Los Baños), 237–248.

[B57] Smita S. Katiyar A. Pandey D. M. Chinnusamy V. Archak S. Bansal K. C. (2013). Identification of conserved drought stress responsive gene-network across tissues and developmental stages in rice. *Bioinformation* 9 72–78. 10.6026/97320630009072 23390349 PMC3563401

[B58] Song Y. Ai C. Jing S. Yu D. (2010). Research progress on functional analysis of rice WRKY genes. *Rice Sci.* 17 60–72.

[B59] Teng S. Keurentjes J. Bentsink L. Koornneef M. Smeekens S. (2005). Sucrose-specific induction of anthocyanin biosynthesis in *Arabidopsis* requires the MYB75/PAP1 gene. *Plant Physiol.* 139 1840–1852. 10.1104/pp.105.066688 16299184 PMC1310563

[B60] Thomas R. González M.-C. (2004). Function and regulation of plant invertases: sweet sensations. *Trends Plant Sci.* 9 606–613. 10.1016/j.tplants.2004.10.009 15564128

[B61] Todaka D. Shinozaki K. Yamaguchi-Shinozaki K. (2015). Recent advances in the dissection of drought-stress regulatory networks and strategies for development of drought-tolerant transgenic rice plants. *Front. Plant Sci.* 6:84. 10.3389/fpls.2015.00084 25741357 PMC4332304

[B62] Toshio I. Akira W. Akiho Y. Michio H. (1991). Aldolase—an important enzyme in controlling the ribulose 1,5-bisphosphate regeneration rate in photosynthesis. *Plant Cell Physiol.* 32 1083–1091.

[B63] Vassey T. L. Quick W. P. Sharkey T. D. Stitt M. (1991). Water stress, carbon dioxide, and light effects on sucrosephosphate synthase activity in *Phaseolus vulgaris*. *Physiol. Plant.* 81 37–44.

[B64] Wang W. Zhang J. Yang J. Zhu Q. (2004). Effect of water stress on metabolism of stored carbohydrate of stem and yield in rice grown under unfavorable-delayed senescence. *Acta Agron. Sin.* 30 196–204.

[B65] Wang Z. Y. Snyder G. W. Esau B. D. Portis A. R. Ogren W. L. (1992). Species-dependent variation in the interaction of substrate-bound ribulose-1,5-bisphosphate carboxylase/oxygenase (rubisco) and rubisco activase. *Plant Physiol.* 100 1858–1862. 10.1104/pp.100.4.1858 16653209 PMC1075876

[B66] Wilkins O. Bräutigam K. Campbell M. M. (2010). Time of day shapes *Arabidopsis* drought transcriptomes. *Plant J.* 63 715–727. 10.1111/j.1365-313X.2010.04274.x 20553421

[B67] Wingler A. Lea P. J. Quick W. P. Leegood R. C. (2000). Photorespiration: metabolic pathways and their role in stress protection. *Philos. Trans. R. Soc.* 355 1517–1529. 10.1098/rstb.2000.0712 11128005 PMC1692872

[B68] Yamada S. Komori T. Hashimoto A. Kuwata S. Imaseki H. Kubo T. (2000). Differential expression of plastidic aldolase genes in Nicotiana plants under salt stress. *Plant Sci.* 154 61–69. 10.1016/s0168-9452(00)00188-6 10725559

[B69] Yang J. Zhang J. Wang Z. Zhu Q. Liu L. (2003). Activities of enzymes involved in sucrose-to-starch metabolism in rice grains subjected to water stress during filling. *Field Crops Res.* 81 69–81.

[B70] Yang J. C. Zhang J. H. Wang Z. Q. Xu G. Zhu Q. (2001). Activities of key enzymes in sucrose-to-starch conversion in wheat grains subjected to water deficit during grain filling. *Plant Physiol.* 135 1621–1629. 10.1104/pp.104.041038 15235118 PMC519076

[B71] Yang Y. (2009). *Isolation and Identification of a Salt-Stress Responsive gene FBPA.* Master’s thesis. Jinan: Shandong University.

[B72] Yousfi S. Márquez A. J. Betti M. Araus J. L. Serret M. D. (2016). Gene expression and physiological responses to salinity and water stress of contrasting durum wheat genotypes. *J. Integr. Plant Biol.* 58 48–66. 10.1111/jipb.12359 25869057

[B73] Yu L. Liu H. Shao X. Yu F. Wei Y. Ni Z. (2016). Effects of hot air and methyl jasmonate treatment on the metabolism of soluble sugars in peach fruit during cold storage. *Postharvest Biol. Technol.* 113 8–16.

[B74] Yu P. Liu S. Han K. Guan S. Zhou D. (2017). Conversion of cropland to forage land and grassland increases soil labile carbon and enzyme activities in northeastern China. *Agric. Ecosyst. Environ.* 245 83–91.

[B75] Yue C. Wang X. Zhou L. He Y. Wang D. Qi Y. (2015). Rubisco activase is also a multiple responder to abiotic stresses in rice. *PLoS One* 10:e0140934. 10.1371/journal.pone.0140934 26479064 PMC4610672

[B76] Zhang C. Liu J. Zhao T. Gomez A. Li C. Yu C. (2016). A drought-inducible transcription factor delays reproductive timing in rice. *Plant Physiol.* 171 334–343. 10.1104/pp.16.01691 26945049 PMC4854678

[B77] Zhang L. Yu S. Zuo K. Luo L. Tang K. (2012). Identification of gene modules associated with drought response in rice by network-based analysis. *PLoS One* 7:e33748. 10.1371/journal.pone.0033748 22662107 PMC3360736

[B78] Zhang X. Wang X. Wang X. Xia G. Pan Q. Fan R. (2006). A shift of phloem unloading from symplasmic to apoplasmic pathway is involved in developmental onset of ripening in grape berry. *Plant Physiol.* 142 220–232. 10.1104/pp.106.081430 16861573 PMC1557625

[B79] Zhao H. Zhang B. Jia Y. Wang Z. Sun B. Gu H. (2017). Effect of drought stress at booting stage on grain nitrogen formation and yield of rice in cold region. *J. Northeast Agric. Univ.* 48 1–10.

