# Supplementary material for: Photosynthetic Carbon Fixation and Sucrose Metabolism Supplemented by Weighted Gene Co-expression Network Analysis in Response to Water Stress in Rice With Overlapping Growth Stages
Source: Front Plant Sci. 2022 Apr 21;13:864605. doi: 10.3389/fpls.2022.864605 (PMC9069116; doi:10.3389/fpls.2022.864605)
Supplement: Supplementary file 1 [file Table_1.doc]

Supplementary Table 1 Statistics of transcriptome sequencing results

| Sample | Clean reads | Mapped Reads | | Uniq Mapped Reads | | Multiple Map Reads | |
| --- | --- | --- | --- | --- | --- | --- | --- |
| SJ_0 | 46,272,717 | 38,847,665 | (83.95%) | 37,842,955 | (81.78%) | 1,004,710 | (2.17%) |
| SJ_3 | 45,656,247 | 38,236,563 | (83.75%) | 37,408,879 | (81.94%) | 827,684 | (1.81%) |
| SJ_6 | 45,318,849 | 38,341,278 | (84.60%) | 37,531,861 | (82.82%) | 809,417 | (1.79%) |
| SJ_9 | 42,238,390 | 35,259,890 | (83.48%) | 34,355,404 | (81.34%) | 904,486 | (2.14%) |
| SJ_12 | 45,155,951 | 38,282,705 | (84.78%) | 37,507,652 | (83.06%) | 775,053 | (1.72%) |
| DN_0 | 43,498,603 | 36,052,087 | (82.88%) | 35,240,051 | (81.01%) | 812,036 | (1.87%) |
| DN_3 | 44,023,155 | 35,884,224 | (81.51%) | 33,646,486 | (76.43%) | 2,237,738 | (5.08%) |
| DN_6 | 46,699,885 | 38,580,068 | (82.61%) | 37,803,402 | (80.95%) | 776,665 | (1.66%) |
| DN_9 | 44,996,960 | 37,247,391 | (82.78%) | 36,572,908 | (81.28%) | 674,483 | (1.50%) |
| DN_12 | 44,819,541 | 37,368,412 | (83.38%) | 36,600,146 | (81.66%) | 768,266 | (1.71%) |

Note: SJ_0: SJ6 before the onset of drought stress; SJ_3: SJ6 under drought stress for 3 days; SJ_6: SJ6 under drought stress for 6 days; SJ_9: SJ6 under drought stress for 9 days; SJ_12: SJ6 under 12 days of drought stress; DN425 numbering rules are the same as SJ6.
