# Supplementary material for: Photosynthetic Carbon Fixation and Sucrose Metabolism Supplemented by Weighted Gene Co-expression Network Analysis in Response to Water Stress in Rice With Overlapping Growth Stages
Source: Front Plant Sci. 2022 Apr 21;13:864605. doi: 10.3389/fpls.2022.864605 (PMC9069116; doi:10.3389/fpls.2022.864605)
Supplement: Supplementary file 2 [file Table_2.doc]

Supplementary Table 2 Contribution of net photosynthetic rate and sucrose metabolism related enzyme activities to leaves sucrose content

| T | TF | DC | IC | | | | DeC |
| --- | --- | --- | --- | --- | --- | --- | --- |
|  |  |  | Pn | VIN | SuSase | SPS | R^2^ |
| A0 | Pn | 0.456 | —— | -0.245 | -1.313 | 1.845 | 0.470 |
|  | VIN | -0.448 | 0.250 | —— | -0.338 | 1.288 | -0.874 |
|  | SuSase | -2.006 | 0.299 | -0.075 | —— | 1.885 | -4.433 |
|  | SPS | 2.172 | 0.388 | -0.265 | -1.740 | —— | -2.311 |
| A1 | Pn | 0.716 | —— | -0.493 | 0.929 | -1.004 | -0.302 |
|  | VIN | 1.921 | -0.184 | —— | 2.312 | -3.316 | -0.874 |
|  | SuSase | 4.682 | 0.142 | 0.948 | —— | -4.827 | -13.067 |
|  | SPS | -4.975 | 0.145 | 1.280 | 4.543 | —— | -34.630 |
| A2 | Pn | -0.054 | —— | -0.070 | -0.182 | 0.603 | -0.035 |
|  | VIN | 0.313 | 0.012 | —— | 0.066 | 0.091 | 0.203 |
|  | SuSase | -0.271 | -0.036 | -0.076 | —— | 1.051 | -0.436 |
|  | SPS | 1.156 | -0.028 | 0.025 | -0.247 | —— | 0.757 |
| A3 | Pn | 2.171 | —— | 0.211 | -1.620 | -0.051 | -1.624 |
|  | VIN | -0.411 | -1.116 | —— | 1.678 | -0.057 | -0.247 |
|  | SuSase | -1.973 | 1.782 | 0.350 | —— | 0.031 | -4.645 |
|  | SPS | -0.142 | 0.782 | -0.166 | 0.437 | —— | -0.279 |
| B0 | Pn | 1.631 | —— | -0.367 | 1.309 | -1.637 | 0.394 |
|  | VIN | -0.546 | 1.097 | —— | 0.996 | -0.930 | -0.973 |
|  | SuSase | 1.547 | 1.381 | -0.352 | —— | -1.609 | 0.596 |
|  | SPS | -1.722 | 1.550 | -0.295 | 1.445 | —— | -6.336 |
| B1 | Pn | 0.224 | —— | 0.066 | 0.074 | -0.108 | 0.064 |
|  | VIN | 0.481 | 0.030 | —— | 0.128 | -0.074 | 0.313 |
|  | SuSase | 1.051 | 0.016 | 0.059 | —— | -0.255 | 0.725 |
|  | SPS | -0.289 | 0.084 | 0.123 | 0.928 | —— | -0.573 |
| B2 | Pn | -0.030 | —— | -0.064 | -1.260 | 1.895 | -0.033 |
|  | VIN | 0.578 | 0.003 | —— | 0.530 | -0.547 | 0.319 |
|  | SuSase | -1.681 | -0.022 | -0.182 | —— | 2.443 | -4.700 |
|  | SPS | 2.471 | -0.023 | -0.128 | -1.662 | —— | -2.854 |
| B3 | Pn | 3.739 | —— | -0.109 | -1.470 | -1.602 | -9.810 |
|  | VIN | 0.339 | -1.205 | —— | 1.456 | -0.131 | 0.197 |
|  | SuSase | -2.014 | 2.728 | -0.245 | —— | -0.584 | -3.593 |
|  | SPS | -1.840 | 3.256 | 0.024 | -0.640 | —— | -6.330 |

注：T：Treatments；TF：Trait factors；DC：Direct contribution；IC：Indirect contribution；DeC：Decision coefficient；Pn：Net photosynthetic rate
