# Supplementary material for: Photosynthetic Carbon Fixation and Sucrose Metabolism Supplemented by Weighted Gene Co-expression Network Analysis in Response to Water Stress in Rice With Overlapping Growth Stages
Source: Front Plant Sci. 2022 Apr 21;13:864605. doi: 10.3389/fpls.2022.864605 (PMC9069116; doi:10.3389/fpls.2022.864605)
Supplement: Supplementary file 3 [file Table_3.doc]

Supplementary Table 3 Primer design for Realtime PCR of DEGs

| Gene ID | Groups | Forward primers (5'-3') | Reverse primers (5'-3') |
| --- | --- | --- | --- |
| LOC_Os04g59440 | DN_0vsDN_9 | CTATTAGCGACTGCATGCTTTG | CAAGGTCAGACACTACCATCAT |
| LOC_Os01g03310 | SJ_0vsSJ_12 | GAGATGGTGGACTCGTGGTC | TTAGTTGTGCATTTCAGGTGTG |
| LOC_Os07g05360 | SJ_0vsSJ_12 | AGAGTGAGTGATCAGTTGAGTG | CTATGATCAAAGATGGCCTTGC |
| LOC_Os02g01590 | SJ_0vsSJ_9 | GCCTAGACTCCTCTCTTCTTCT | GATAAAATTCTGAAGGCCAGGC |
| LOC_Os06g04230 | SJ_0vsSJ_9 | ACACACAAATCAAACCACACTG | GGATCTTGTCCATGATCAAGAA |
| LOC_Os04g55710 | SJ_0vsSJ_9 | TGTTCGTGGGCTAGAATTAGTG | CGCCATCATCGTCATTATTCAA |
| LOC_Os11g44810 | SJ_0vsSJ_6 | AGTAGCTTGCTGTCTCTGTTAG | TAGGGAAACACATGGCAGTATT |
| LOC_Os01g13690 | DN_0vsDN_6 | AACGTGCCGAGTATATATGTGT | TCATACAGTACTGATACACCGC |
| LOC_Os08g03290 | internal control gene | CCAAGACCCAGTAGAATCCTTT | CTCAGGTCCATATCATCAGCAT |
| LOC_Os05g06770 | internal control gene | TTGCTCTGATCTGATGCTTTTC | CCAAGAGGCTACTACTCAAAGT |
