# Supplementary figures and images for: Photosynthetic Carbon Fixation and Sucrose Metabolism Supplemented by Weighted Gene Co-expression Network Analysis in Response to Water Stress in Rice With Overlapping Growth Stages
Source: Front Plant Sci. 2022 Apr 21;13:864605. doi: 10.3389/fpls.2022.864605 (PMC9069116; doi:10.3389/fpls.2022.864605)

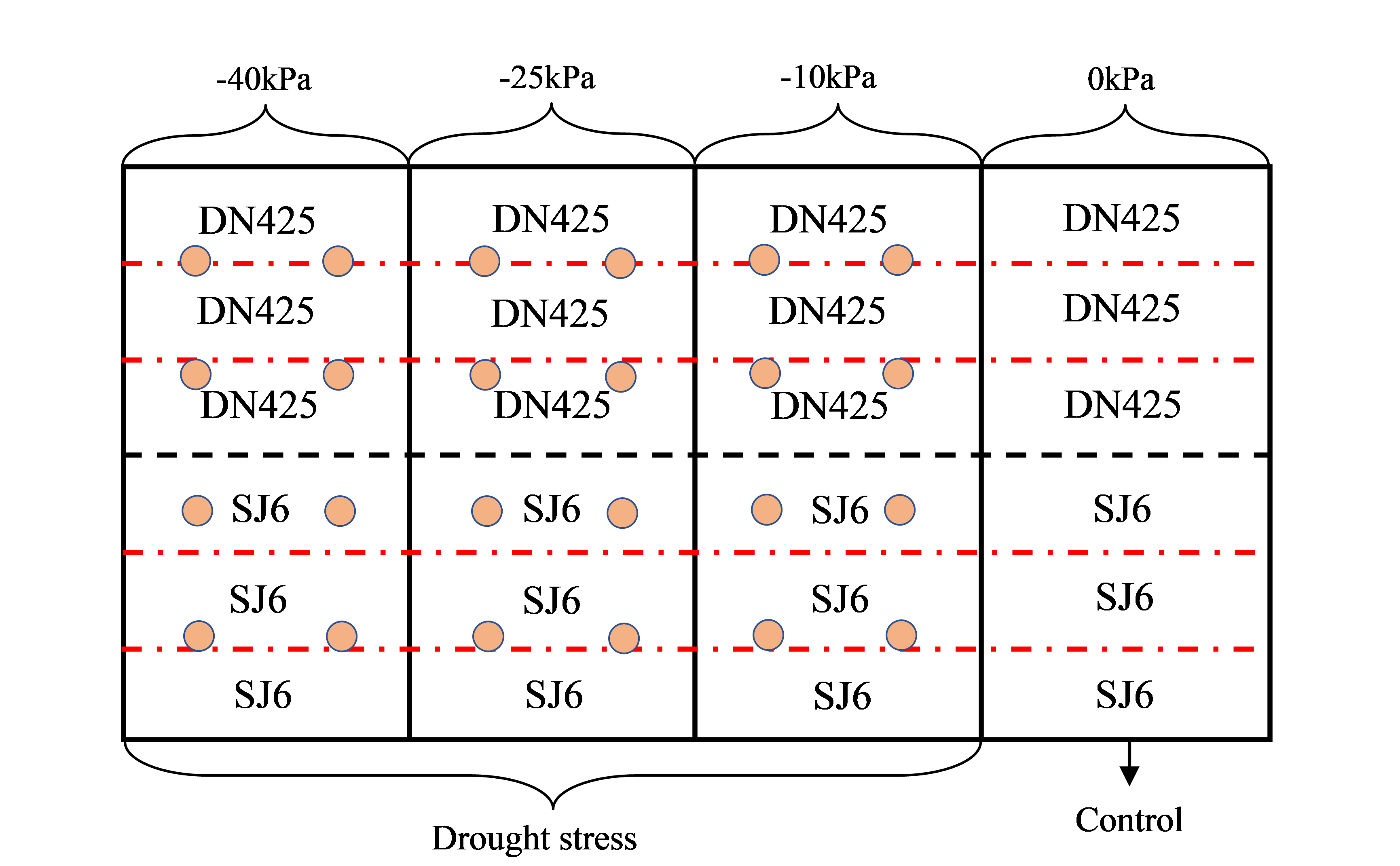

Supplement: Supplementary Figure 1 — Field map of the experimental design. The red dotted line represents the separation between repetitions, and the black dotted line represents the partition between varieties. The circle represents the position of the soil tensiometer. The black line represents the soil ridge for separating treatments. [file Image_1.png]

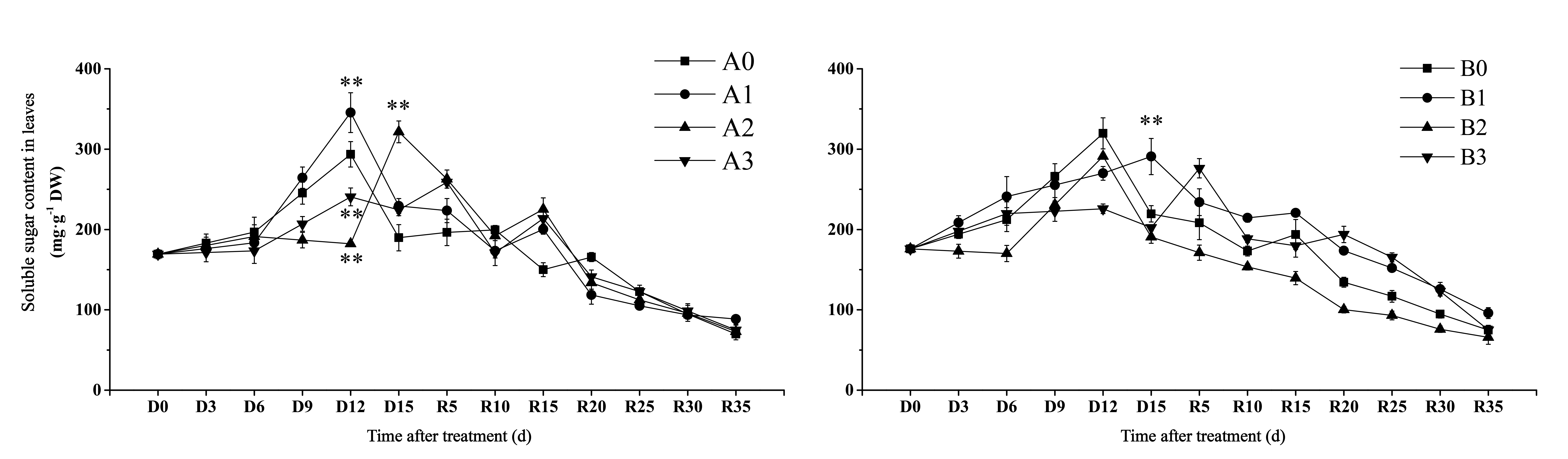

Supplement: Supplementary Figure 2 — Effect of drought stress on the soluble sugar content of growth period overlapping rice leaves at the jointing-booting stage. **Represent significance at P < 0.01, respectively. [file Image_2.jpg]

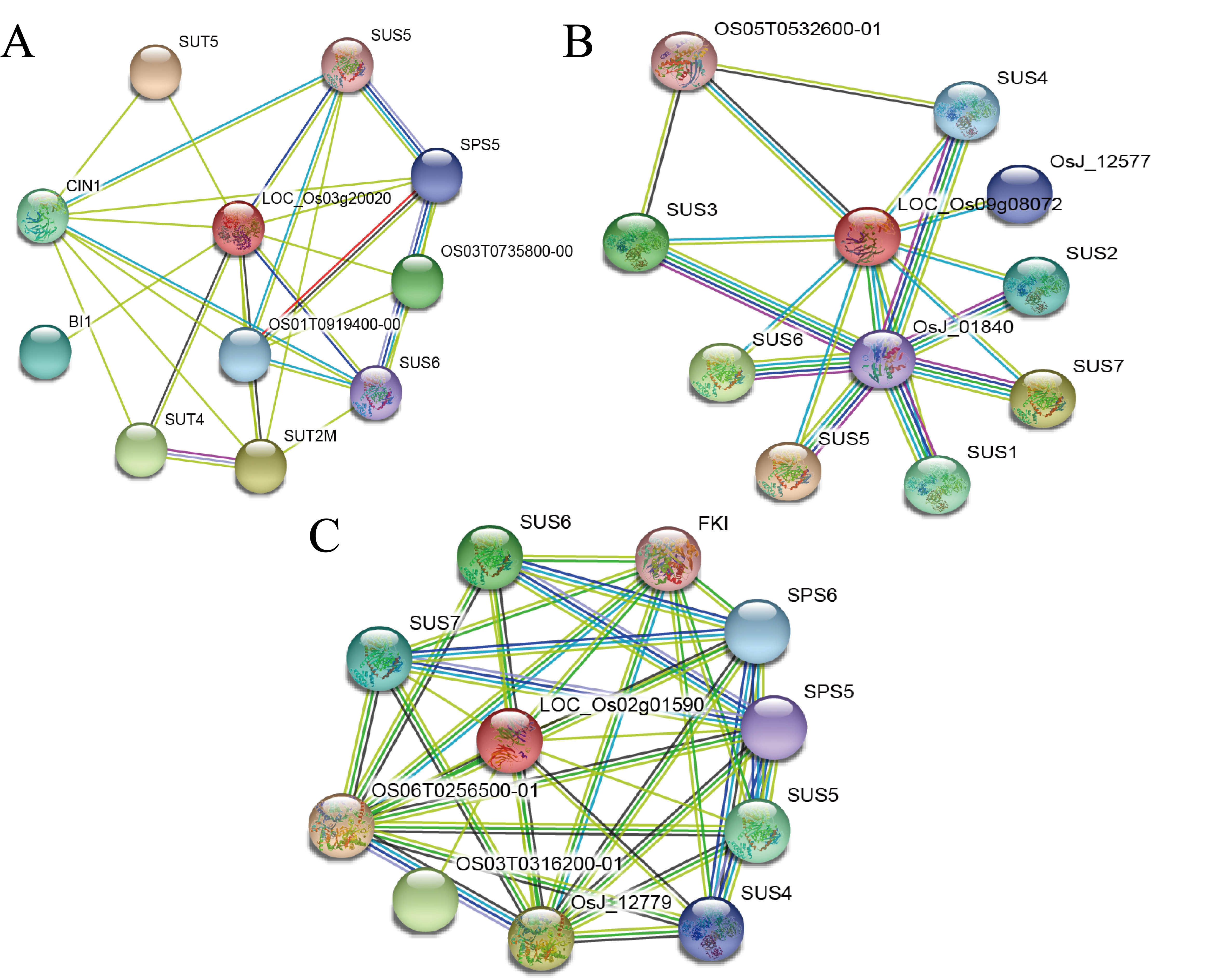

Supplement: Supplementary Figure 3 — Prediction of invertase protein interaction network. (A) LOC_Os03g20020, (B) LOC_Os09g08072, and (C) LOC_Os02g01590. [file Image_3.png]

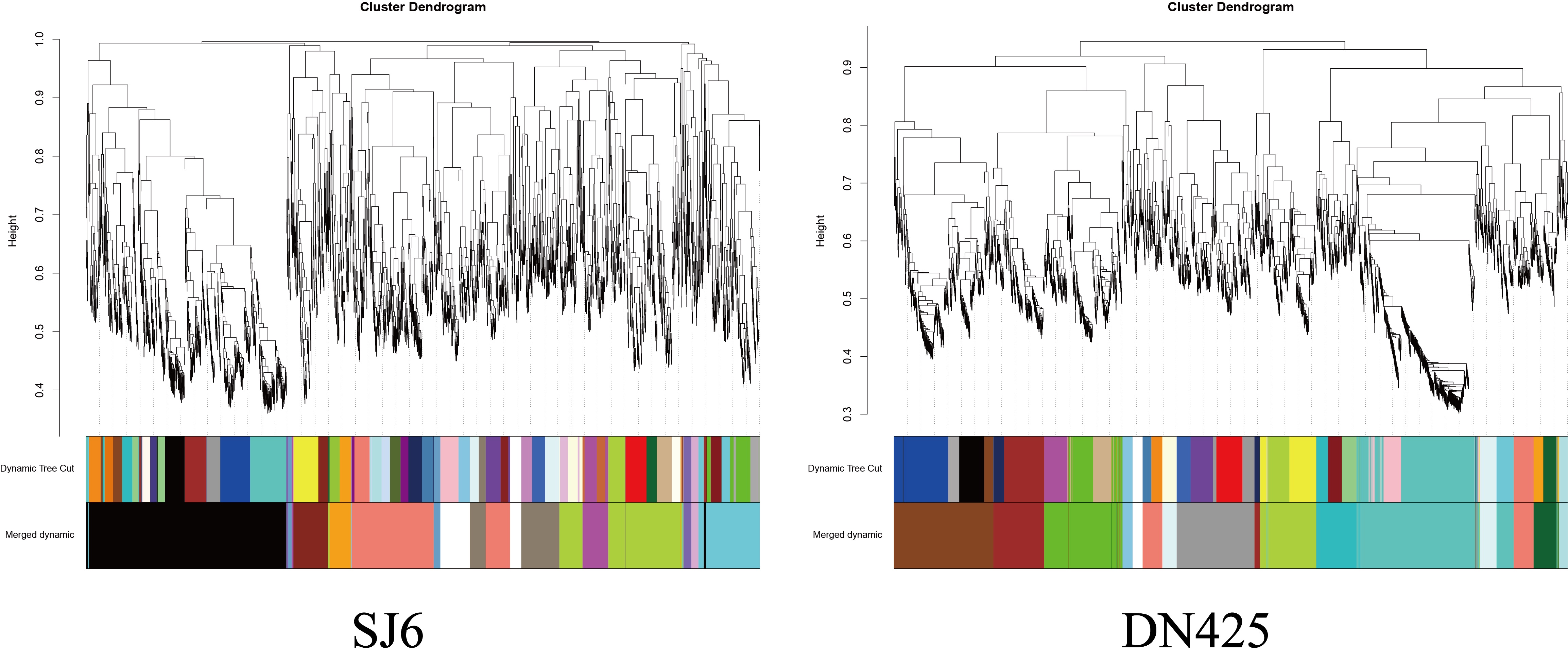

Supplement: Supplementary Figure 4 — Gene merging module cluster tree. [file Image_4.jpg]

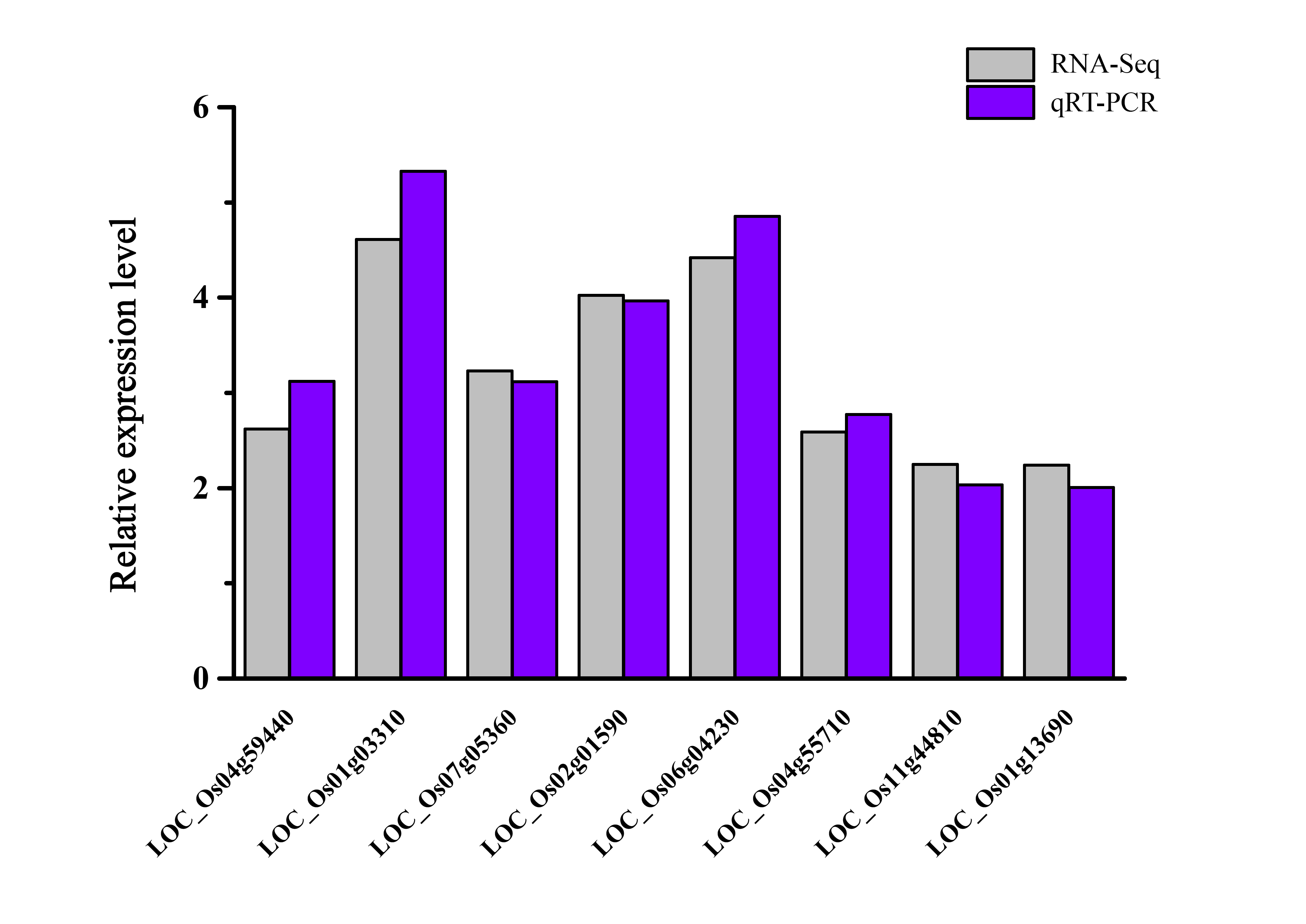

Supplement: Supplementary Figure 5 — DEGs between RNA-Seq and qRT-PCR. [file Image_5.jpg]

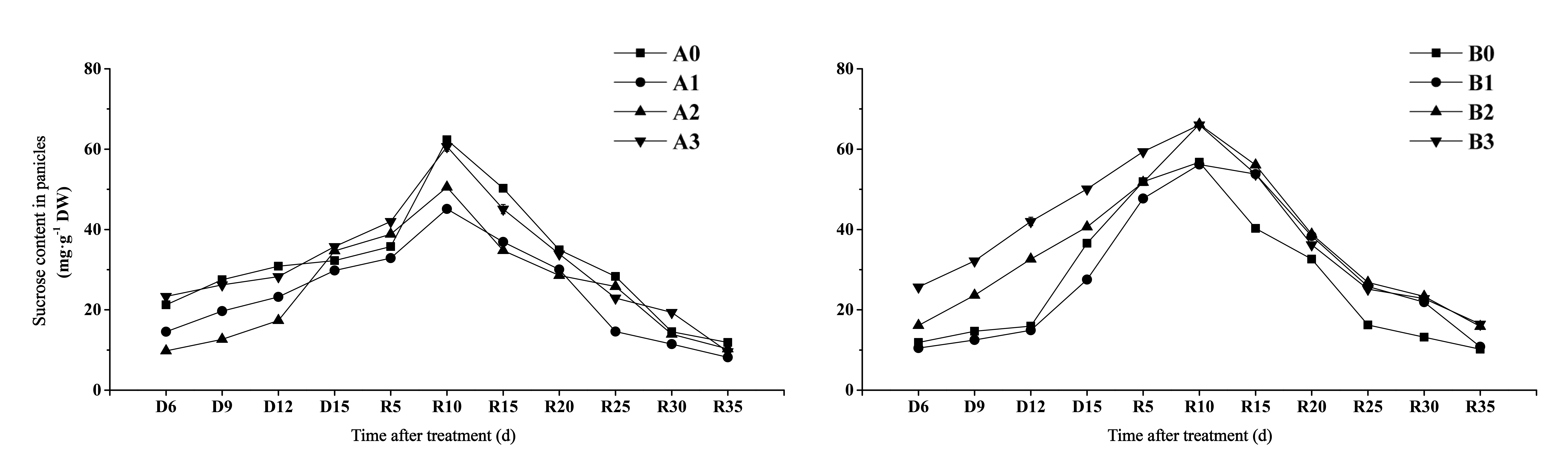

Supplement: Supplementary Figure 6 — Effect of drought stress on sucrose content of growth period overlapping rice panicles at the jointing-booting stage. [file Image_6.jpg]
